# Supplementary material for: Association between health indicators of maternal adversity and the rate of infant entry to local authority care in England: a longitudinal ecological study
Source: BMJ Open. 2020 Aug 13;10(8):e036564. doi: 10.1136/bmjopen-2019-036564 (PMC7430489; doi:10.1136/bmjopen-2019-036564)
Supplement: Supplementary data [file bmjopen-2019-036564supp001.pdf]

Supplementary appendix

Contents

Figure A1: An ecological framework of child maltreatment 2
Figure A2: A flow diagram of the Children Looked After (CLA) cohort selection 3
Missing data: CLA return 3
Figure A3: A flow diagram of the Hospital Episode Statistics Admitted Patient Care (HES APC) cohort selection 4
Missing data: HES APC 4
Table A1: Censored values in measures derived from HES APC 5
Table A2: ICD-10 code lists to identify an adversity-related hospital admission (ARA) 6
Figure A4: The distribution of rate of infant entry to care over the study period 7
Table A3 - Linear mixed-effect modelling results (Models 1-4) 8
Table A4 – Linear mixed-effect modelling results (Model 5) 10
Table A5: Conditional coefficient estimates for percentage of live births with maternal history of adversity-related hospital admissions, by financial year (Model 5) 11

Pearson RJ, *et al. BMJ Open* 2020; 10:e036564. doi: 10.1136/bmjopen-2019-036564

Figure A1: An ecological framework of child maltreatment

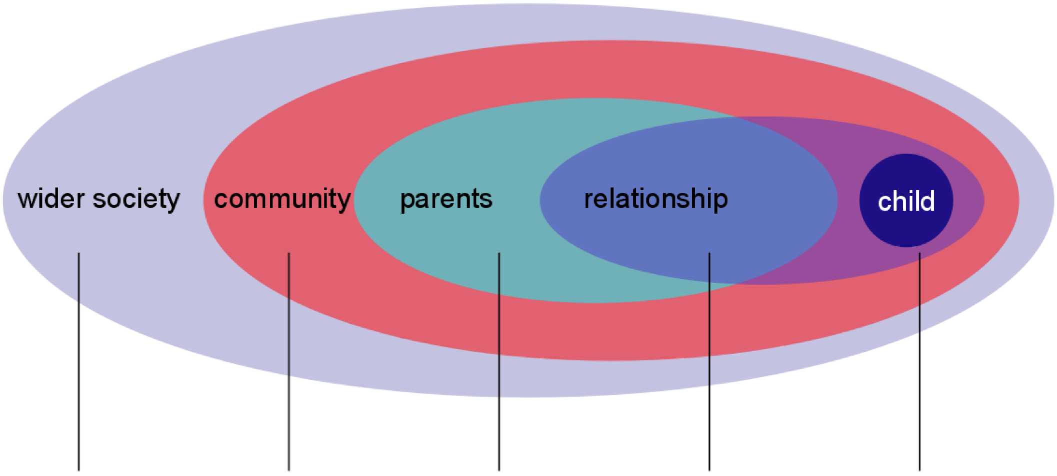

wider society
- social and cultural norms of child maltreatment
- policies and laws
- systematic inequality
- resource allocation

community
- residential instability
- community violence
- high unemployment
- poor school and pre-school opportunities
- lack of parenting programmes
- poor access to healthcare and social services

parental factors:
- single parent
- young age
- experienced abuse or neglect in childhood
- physical or developmental disability
- mental ill health
- substance misuse
- unsupported and/or isolated

environmental factors:
- domestic abuse
- non-biological adult living in the house
- lack of resources

relationship
- parental perceptions of child's intentions and development
- parenting attitudes and practice
- cultural beliefs and values

child
- physical or developmental disability
- behavioural problems
- incarceration
- young age
- premature birth
- low birthweight

Recognising the ecological and interconnected nature of risk-factors for child maltreatment at different ecological levels, many have applied an ecological framework to this field of study over the years.[1–3] The ecological framework below builds upon previous diagrams and incorporates evidence from several systematic reviews looking at risk-factors for child maltreatment. [4–6]

Figure A2: A flow diagram of the Children Looked After (CLA) cohort selection

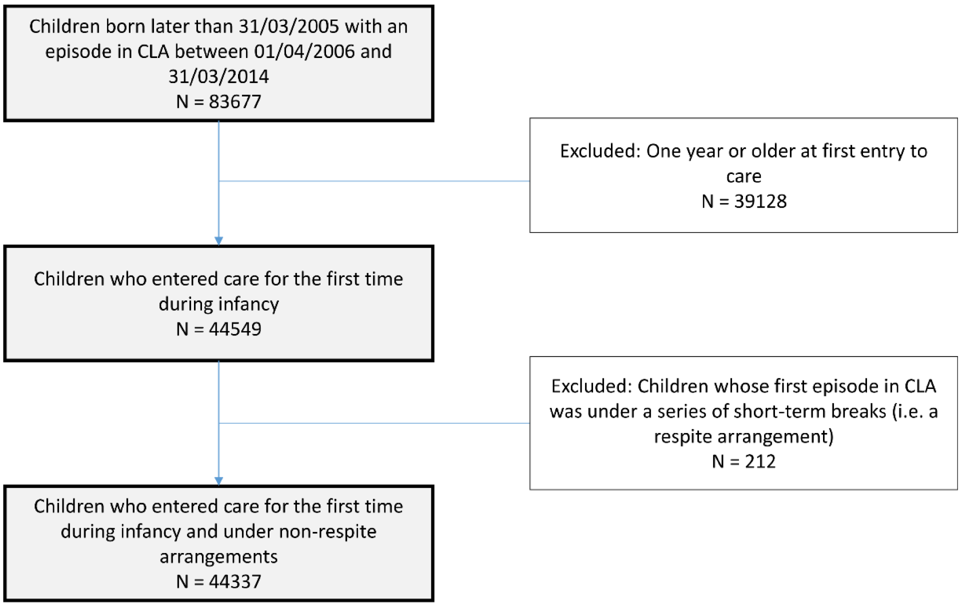

Missing data: CLA return

Adhering to the Department for Education's statistical disclosure rules, we censored any counts less than 10 before outputting them from our secure data environment. Of our 1048 LA counts of infants entering care, 60 (5.7%) had to be censored before we could output them from UCL's secure data environment. We imputed these censored values with a randomly selected number from 0 to 9 before analysis (random seed set to 1234).

Figure A3: A flow diagram of the Hospital Episode Statistics Admitted Patient Care (HES APC) cohort selection

We derived two cohorts using HES APC (please note that the extract of HES APC used only included individuals with dates of birth available):

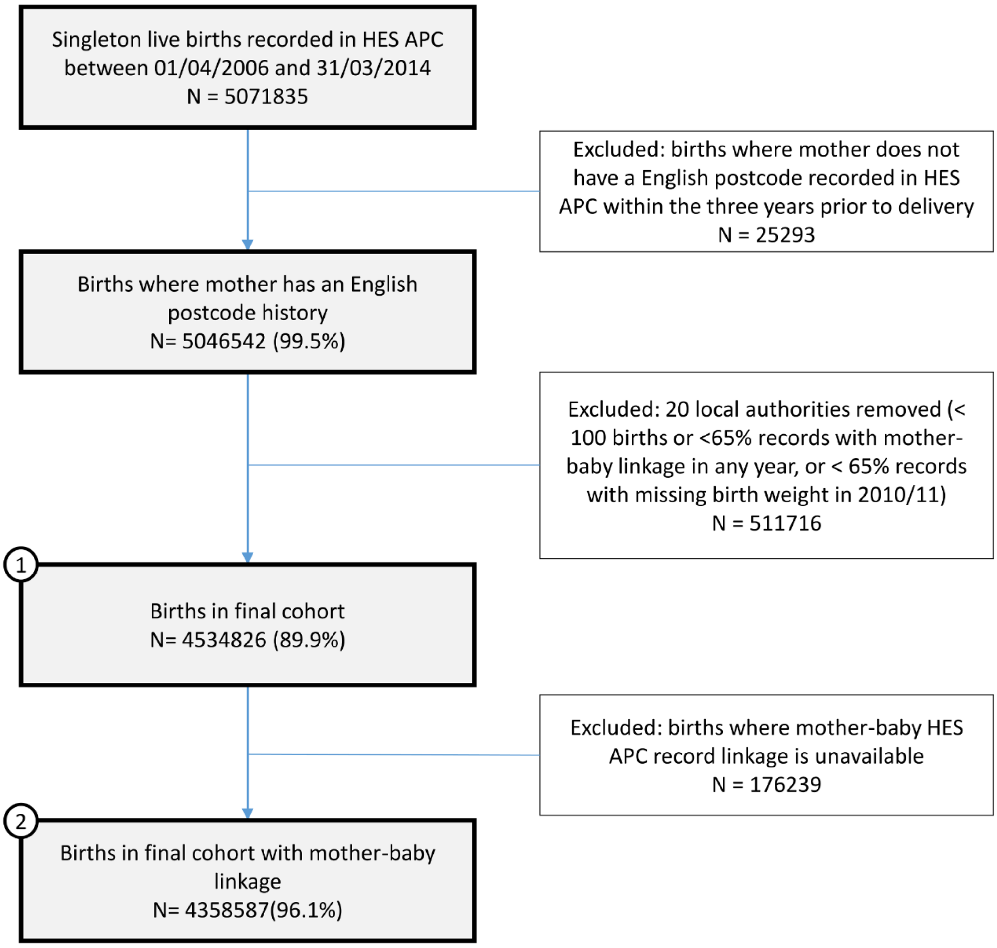

Missing data: HES APC

Adhering to the Department for Health and Social Care's statistical disclosure rules, we censored any values derived from HES APC where the underlying counts were less than five before outputting them from our secure data environment. After outputting, we imputed censored counts with a randomly selected number from 0 to 4 (random seed set to 1234).

Table A1: Censored values in measures derived from HES APC

Explanatory measure	Number of values	Number of values less than five (%)
% of live births with maternal history of ARA	1048	0
% of live births where mother < 20 years old	1048	0
% of live births where maternal LSOA history within 10% most deprived LSOAs in England	1048	24 (2.29%)
% of live births where child has a complex chronic condition	1048	1 (0.10%)
% of live births with low birth weight	1048	0

Pearson RJ, et al. BMJ Open 2020; 10:e036564. doi: 10.1136/bmjopen-2019-036564

Table A2: ICD-10 code lists to identify an adversity-related hospital admission (ARA)

ICD-10 codes	Source
Mental Health disorders:	
Behavioural and emotional disorder<br>F90-F95, F98	Nilsson, 2017
Neurotic, stress-related, and somatoform disorder<br>F40-F45, F48	
Schizophrenia , Personality disorder, or Bipolar disorder<br>F20, F30-F33, F60-F62	
Depressive episode or disorder<br>F32-F33	
Substance use disorder<br>F10-F16, F18-F19	
Other mental health disorder<br>F00-F09, F21-F25, F28, F34, F38-F39, F50-F55, F59, F63-F66, F68-F73, F79-F84, F88-F89, F99	
Intentional self-harm<br>X60-X84	
History of self-harm<br>Z91.5 (we also included Y87.0 – sequelae of intentional self-harm)	Herbert, 2015
Substance misuse:	
Alcohol misuse<br>E51.2, T51.0, X45.0-X45.2, X45.4, X45.6, X45.8, X45.9, X65.0-X65.2, X65.4, X65.5, X65.6, X65.8, X65.9, Y15.0, Y15.2, Y15.4, Y15.8, Y15.9, Y90.0-Y91.3, Y91.9, Z50.2, Z71.4, Z72.1	Fone, 2016
Alcohol and other substance misuse<br>E24.4, F10-F16, F18-F19, G31.2, G62.1, G72.1, I42.6, K29.2, K70.0-K70.9, K85.2, K86.0, O35.4, R78.0-R78.5, T36-T50 (we did not exclude T50.6, however), X40-X44, X46-X49, X69, Y10-Y14, Y16-Y19, Z04.0, Z50.3, Z71.5, Z72.2, T51	Herbert, 2015
Exposure to violence:	
Assault<br>X85-X99, Y01-Y05, Y08-Y09	Herbert, 2015
Maltreatment<br>T73-T74, Y06-Y07	
Injury: undetermined cause<br>Y20-Y34, Z04.5 (we excluded Z04.8)	

Pearson RJ, *et al. BMJ Open* 2020; 10:e036564. doi: 10.1136/bmjopen-2019-036564

Figure A4: The distribution of rate of infant entry to care over the study period

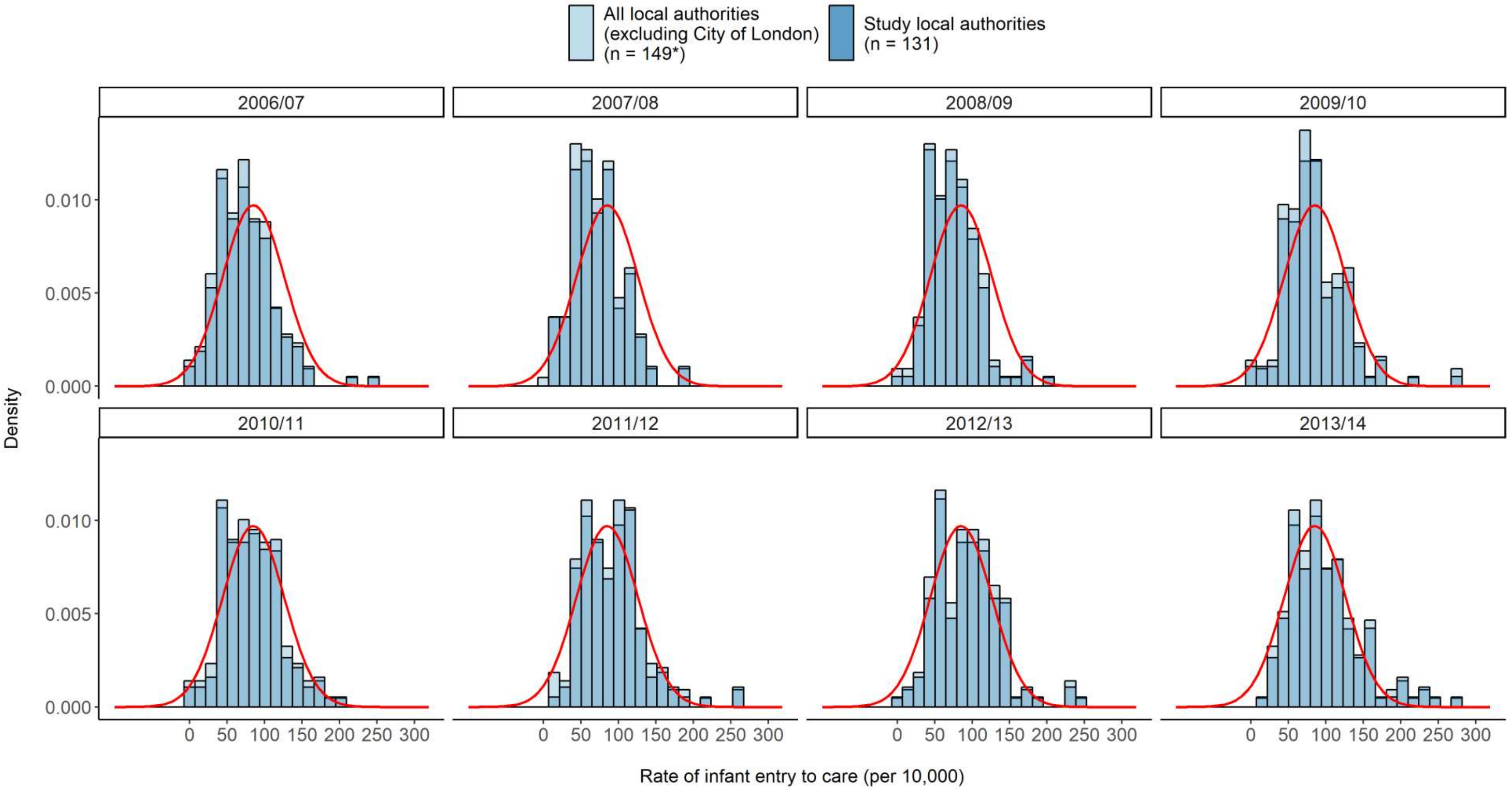

* There were 151 local authorities in 2006/07; however, the Isles of Scilly did not have any children in their care over the study period.
Data source: Children Looked After return, Department for Education.

Table A3 - Linear mixed-effect modelling results (Models 1-4)

Model parameter	Model 1		Model 2		Model 3		Model 4		Type of model parameter
	Estimate	95% C.I.	Estimate	95% C.I.	Estimate	95% C.I.	Estimate	95% C.I.	
Time (e.g. financial year Apr-March)	3.99	3.15 to 4.84	2.46	1.29 to 3.64	2.53	1.35 to 3.71	2.39	1.25 to 3.53	fixed-effect
% of live births with maternal history of ARA			(within-LA) 2.44 (between-LA) 11.63	1.10 to 3.78 8.94 to 14.31	2.33 3.89	0.98 to 3.68 0.79 to 6.98	(overall) 2.56	1.31 to 3.82	fixed-effect
% of live births where mother < 20 years old					6.77	4.50 to 9.03	7.25	5.22 to 9.28	fixed-effect
% of live births where maternal postcode history within 10% most deprived LSOAs in England					0.01	-0.35 to 0.37	0.04	-0.31 to 0.39	fixed-effect
% of live births where child has a complex chronic condition					13.42	4.55 to 22.30	14.22	5.51 to 22.93	fixed-effect
% of live births with low birth weight					1.28	-2.93 to 5.48	1.00	-3.16 to 5.17	fixed-effect
LA population size (000s)					-0.08	-0.14 to -0.02	-0.08	-0.14 to -0.02	fixed-effect
% of dependent child households with lone parent					1.87	0.65 to 3.09	1.96	0.76 to 3.17	fixed-effect
Rate of violent crime (per 100 LA residents)					6.10	-3.12 to 15.32	5.05	-3.88 to 13.98	fixed-effect
LA – random intercept (sd)	29.25		24.50		20.56		20.58		random-effect

Time (financial year) – random slope (sd)	3.91		3.78		3.78		3.78		random-effect
Correlation between random intercept and random slope	0.14		-0.11		-0.45		-0.45		random-effect
Residual error	19.43		19.37		19.37		19.37		residual

ARA = adversity-related hospital admission; **LA** = local authority; **LSOA** = lower-layer super output area; **sd** = standard deviation.

Table A4 – Linear mixed-effect modelling results (Model 5)

Model parameter	Estimate	95% C.I.	Type of model parameter
Time (financial year) (where average maternal ARA = 0)	0.78	-0.54 to 2.10	fixed-effect
% of live births with maternal history of ARA (in 2006/07)	0.16	-1.93 to 2.25	fixed-effect
Effect of unit increase in time on average % of live births with maternal history of ARA (or vice versa)	0.44	0.15 to 0.72	fixed-effect
% of live births where mother < 20 years old	8.29	6.23 to 10.35	fixed-effect
% of live births where maternal postcode history within 10% most deprived LSOAs in England	0.06	-0.30 to 0.41	fixed-effect
% of live births where child has a complex chronic condition	14.19	5.37 to 23.01	fixed-effect
% of live births with low birth weight	0.60	-3.62 to 4.82	fixed-effect
LA population size (000s)	-0.08	-0.15 to -0.02	fixed-effect
% of dependent child households with lone parent	1.92	0.70 to 3.14	fixed-effect
Rate of violent crime (per 100 LA residents)	5.20	-3.86 to 14.25	fixed-effect
Local Authority – random intercept (sd)	18.55		random-effect
Residual error	21.35		residual

ARA = adversity-related hospital admission; **LA** = local authority; **LSOA** = lower-layer super output area; **sd =** standard deviation.

Pearson RJ, *et al. BMJ Open* 2020; 10:e036564. doi: 10.1136/bmjopen-2019-036564

Table A5: Conditional coefficient estimates for percentage of live births with maternal history of adversity-related hospital admissions, by financial year (Model 5)

Measure	Financial year	Conditional coefficient estimate	95% C.I.
Effect of a 1% point increase in % of live births with maternal history of ARA in…	2006/07	0.13	-2.01 to 2.28
	2007/08	0.57	-1.32 to 2.48
	2008/09	1.01	-0.65 to 2.69
	2009/10	1.45	-0.03 to 2.93
	2010/11	1.89	0.59 to 3.21
	2011/12	2.33	1.13 to 3.53
	2012/13	2.77	1.61 to 3.93
	2013/14	3.21	2.03 to 4.39

ARA = adversity-related hospital admission.
Note: Created using R package 'interplot' https://cran.r-project.org/web/packages/interplot/index.html.

References

1. Bronfenbrenner U. The Ecology of Human Development. The Educational Forum. 1979.

2. Belsky J. Child maltreatment: An ecological integration. Am Psychol. 1980;

3. Sidebotham P. An ecological approach to child abuse: A creative use of scientific models in research and practice. Child Abus Rev. 2001;10(2):97–112.

4. Stith SM, Liu T, Davies LC, et al. Risk factors in child maltreatment: A meta-analytic review of the literature. Aggression and Violent Behavior. 2009.

5. Mulder TM, Kuiper KC, van der Put CE, Stams G-JJM, Assink M. Risk factors for child neglect: A meta-analytic review. Child Abuse Negl. 2018;77:198–210.

6. Sidebotham P, Heron J, Team AS. Child maltreatment in the “children of the nineties”: a cohort study of risk factors. Child Abuse Negl. 2006;
