# Supplementary material for: Association between health indicators of maternal adversity and the rate of infant entry to local authority care in England: a longitudinal ecological study
Source: BMJ Open. 2020 Aug 13;10(8):e036564. doi: 10.1136/bmjopen-2019-036564 (PMC7430489; doi:10.1136/bmjopen-2019-036564)
Supplement: Author's manuscript [file bmjopen-2019-036564.draft_revisions.pdf]

BMJ Open

BMJ Open is committed to open peer review. As part of this commitment we make the peer review history of every article we publish publicly available.

When an article is published we post the peer reviewers' comments and the authors' responses online. We also post the versions of the paper that were used during peer review. These are the versions that the peer review comments apply to.

The versions of the paper that follow are the versions that were submitted during the peer review process. They are not the versions of record or the final published versions. They should not be cited or distributed as the published version of this manuscript.

BMJ Open is an open access journal and the full, final, typeset and author-corrected version of record of the manuscript is available on our site with no access controls, subscription charges or pay-per-view fees (http://bmjopen.bmj.com).

If you have any questions on BMJ Open's open peer review process please email info.bmjopen@bmj.com

Association between health indicators of maternal adversity and the rate of infant entry to local authority care in England: a longitudinal ecological study

Journal:	*BMJ Open*
Manuscript ID	bmjopen-2019-036564
Article Type:	Original research
Date Submitted by the Author:	20-Dec-2019
Complete List of Authors:	Pearson, Rachel; UCL Great Ormond Street Institute of Child Health, Population, Policy and Practice Research and Teaching Department<br>Jay, Matthew; UCL Great Ormond Street Institute of Child Health, Population, Policy and Practice Research and Teaching Department<br>Wijlaars, Linda; UCL Great Ormond Street Institute of Child Health, Population, Policy and Practice Research and Teaching Department<br>De Stavola, Bianca ; UCL Great Ormond Street Institute of Child Health, Population, Policy and Practice Research and Teaching Department<br>Syed, Shabeer; UCL Great Ormond Street Institute of Child Health, Population, Policy and Practice Research and Teaching Department<br>Bedston, Stuart; Lancaster University, Department for Sociology, Centre for Child and Family Justice Research<br>Gilbert, Ruth; UCL Great Ormond Street Institute of Child Health, Population, Policy and Practice Research and Teaching Department
Keywords:	PUBLIC HEALTH, EPIDEMIOLOGY, Adult psychiatry < PSYCHIATRY, Maternal medicine < OBSTETRICS

SCHOLARONE™ Manuscripts

I, the Submitting Author has the right to grant and does grant on behalf of all authors of the Work (as defined in the below author licence), an exclusive licence and/or a non-exclusive licence for contributions from authors who are: i) UK Crown employees; ii) where BMJ has agreed a CC-BY licence shall apply, and/or iii) in accordance with the terms applicable for US Federal Government officers or employees acting as part of their official duties; on a worldwide, perpetual, irrevocable, royalty-free basis to BMJ Publishing Group Ltd ("BMJ") its licensees and where the relevant Journal is co-owned by BMJ to the co-owners of the Journal, to publish the Work in this journal and any other BMJ products and to exploit all rights, as set out in our licence.

The Submitting Author accepts and understands that any supply made under these terms is made by BMJ to the Submitting Author unless you are acting as an employee on behalf of your employer or a postgraduate student of an affiliated institution which is paying any applicable article publishing charge ("APC") for Open Access articles. Where the Submitting Author wishes to make the Work available on an Open Access basis (and intends to pay the relevant APC), the terms of reuse of such Open Access shall be governed by a Creative Commons licence – details of these licences and which Creative Commons licence will apply to this Work are set out in our licence referred to above.

Other than as permitted in any relevant BMJ Author's Self Archiving Policies, I confirm this Work has not been accepted for publication elsewhere, is not being considered for publication elsewhere and does not duplicate material already published. I confirm all authors consent to publication of this Work and authorise the granting of this licence.

For peer review only

Association between health indicators of maternal adversity and the rate of infant entry to local authority care in England: a longitudinal ecological study

Rachel J. Pearson, MSc [1]

Matthew A. Jay, MSc [1]

Linda Wijlaars, PhD [1]

Bianca L. De Stavola, PhD [1]

Shabeer Syed, MSc[1]

Stuart Bedston, PhD [2]

Ruth Gilbert, MD [1]

1 Population, Policy and Practice Research and Teaching Department
UCL Great Ormond Street Institute of Child Health
30 Guilford Street
University College London, London
WC1N 1EH

2 Centre for Child and Family Justice Research
Department of Sociology
Lancaster University
LA1 4YT

Corresponding author:
Rachel J. Pearson
222 Euston Road,
London,
NW1 2DA
Email: Rachel.Pearson@ucl.ac.uk
Tel: 020 3549 5994

For peer review only - http://bmjopen.bmj.com/site/about/guidelines.xhtml

ABSTRACT

Objective
Infants enter care at varying rates across local authorities (LAs) in England, but evidence is lacking on what is driving these differences. With this ecological study, we aimed to explore the extent to which adversity indicated within women's hospitalisation histories, pre-delivery, explained rate of infant entry into care.

Methods
We used two longitudinal person-level data sets on hospitalisations and entries to care to create annual measures for 131 English LAs, between 2006/07 and 2013/14 (Apr-Mar). We combined these measures by LA and financial year, along with other publicly-available data on LA characteristics. We used linear mixed-effects models to analyse the relationship between the outcome - LA-specific rate of infant entry into care (per 10,000 infants in the LA population) - and LA-specific percentage of livebirths with maternal history of adversity-related hospital admissions (i.e. substance misuse, mental health problems or violence-related admissions in the three years before delivery), adjusted for other predictors for entry into care.

Results
Rate of infant entry into care (mean: 85·16 per 10,000, SD: 41·07) and percentage of livebirths with maternal history of adversity-related hospital admissions (4·62%, 2·44%) varied greatly by LA. Prevalence of maternal adversity accounted for 24% of the variation in rate of entry (95% CI: 14-35%). After adjustment, a percentage point increase in prevalence of maternal adversity - both within and between LAs - was associated with an estimated 2·56 (per 10,000) more infants entering care (1·31-3·82).

Conclusions
Prevalence of maternal adversity before birth helped to explain variation in LA rates of infant entry into care. Preventive interventions are needed to improve maternal well-being before and during pregnancy, and potentially reduce risk of child maltreatment and, therefore, entries to care. Evidence on who to target and data to evaluate change requires linkage between parent-child healthcare data and administrative data from children's social care.

For peer review only - http://bmjopen.bmj.com/site/about/guidelines.xhtml

Strengths and limitations of this study

- This is the first study to examine the relationship between LA variation in rate of entry into care in England and parental health indicators that are associated with diminished parenting capacity.
- In our analysis, we used a breadth of information on mothers and children derived from longitudinal, person-level Hospital Episode Statistics (HES) records and our outcome was derived from the Children Looked After return (CLA) database containing all entries into care in England.
- Our estimates of local authority prevalence of live births with history of maternal adversity-related hospital admission (i.e substance misuse, mental health, or violence- related admissions up to 3 years before delivery) are likely to be underestimated due to poor recording, reporting or recognition of these problems.
- Due to the lack of national data linkages between mother-to-baby linked healthcare data and children's social care outcomes in England, we could not examine whether children born to women with a history of maternal adversity-related hospital admission were more likely to be placed into care during infancy.

For peer review only

For peer review only - http://bmjopen.bmj.com/site/about/guidelines.xhtml

INTRODUCTION

England has experienced unsustainable increases in the number of children in public care in recent years, leaving its children's social care sector overburdened.[1,2] One in five children entering local authority care are infants (aged under one year) and the rate of infant entry into care has increased by almost 20% since 2010/11.[1] Similar patterns and increases in infant entry to care are taking place in other countries including Scotland, the US, New Zealand, and parts of Australia. There is also marked regional variation in rates of entry across each of these settings,[3–7] with limited evidence on what drives differences.[8,9] The most frequently documented entry reason among infants entering care in England is abuse and neglect (i.e. child maltreatment).[1] Maltreatment in infancy is linked to poorer physical, intellectual and behavioural development in childhood and to substance misuse, risky sexual behaviour, domestic violence and abuse, self-harm, and poorer health outcomes later in life.[10,11] Developing our understanding of the drivers of geographic variation in the prevalence of maltreatment and demand for child protection intervention in infancy is, therefore, a key public health issue.

The responsibility for placing infants into care in England rests with local authorities (LAs) who have a statutory duty to safeguard children.[12] Past studies have shown that entry into care is driven by a complex ecological framework of risk factors relating to children and their families, the local community, service thresholds and capacity, and wider society.[13–15] These studies also highlight strong associations between parental adversities - such as mental health problems or deprivation - and child maltreatment or entry into care. Experiences of substance misuse, domestic violence, and mental health problems are common among parents whose children are subject to child protection in England.[16] Parents who experience these forms of adversity are also more likely to have serious and complex healthcare needs,[17] as well as being more likely to struggle to meet their child's needs and to maltreat their child (Figure 1).[13,14] In Western Australia and Manitoba, Canada, analyses of linked record-level administrative health and social care data showed that maternal mental health, substance-related or assault-related hospital admissions were associated with a higher relative risk of child entry into care.[18] Therefore, in this study, we examined the extent to which LA-specific prevalence of health indicators for maternal adversity prior to birth explained variation in rate of infant entry into care among LAs in England. With this work, we aim to inform national and local policy strategies to mitigate long-lasting harm to children arising from serious parental adversity and diminished capacity to parent.

[Insert Figure 1 here – Figure 1: A hypothetical (simplified) pathway from parental adversity to infant entry into care]

(A) Substance misuse, domestic violence and abuse, and mental health problems not only affect day-to-day functioning but can also lead to serious and complex healthcare needs. **(B)** These forms of adversity can also affect the capacity to parent and may result in harm to the unborn child or infant. Also, pregnancy, birth and caring for an infant place additional stress on parents which can exacerbate experiences of adversity. **(C & D)** Where children are at a significant risk of harm, children's social care services have the power to apply for a court order to receive the child into care or may otherwise receive a child into care where to do so is in the child's best interests and the parents do not object.

For peer review only - http://bmjopen.bmj.com/site/about/guidelines.xhtml

As English children's social care data is not yet linked at the child level to longitudinal healthcare data, we conducted an ecological analysis. We have explored the relationship between LA-specific rates of maternal hospitalisation before birth due to substance misuse, domestic violence and abuse, and mental health problems and LA variation in the rate of infant entry into care. We also accounted for a range of other LA-level risk factors for entry into care including LA-specific prevalence of maternal deprivation, births to teenage mothers, and community violence.

METHODS

Study design

This longitudinal, ecological analysis used yearly (Apr-Mar) aggregate measures between 2006/07 and 2013/14 for 131 out of a possible 151 (as at April 2006) English LAs, derived from several data sources. We excluded 20 LAs from our analysis for having too few live births or poor data quality in at least one measure in one or more years (Table 1). All data were de-identified or already publicly available and anonymised, therefore this study did not require ethical approval.

Outcome

Our outcome was the annual incidence rate of first entry into care during infancy (i.e. under one year old) per 10,000 infants resident in the LA. The longitudinal Children Looked After (CLA) dataset, provided by the Department for Education, was used to determine the numerator for each LA, with Office for National Statistics (ONS) population estimates being used to define the denominator.[19] CLA is a statutory data collection collated by the Department for Education and contains record-level information on all children in care in England, based on annual submissions from each LA. CLA includes information on child demographics (e.g. age, sex, ethnicity etc.) and episodes in care (e.g. episode dates, legal status, placement type etc.).[20] We used a longitudinal extract of CLA containing episode-in-care-level information for all children who entered care between 1st April 2005 and 31st March 2014, excluding infants who first entered care under respite arrangements (i.e. an agreed series of short-term breaks, typically provided for children with complex healthcare needs). For further information on this extract, see Appendix page 3.

Explanatory measures

The explanatory measures were selected to capture 'demand-side' risk factors (i.e. local service need) for child maltreatment and entry into care (Table 1). These measures were chosen based upon evidence, [13–15] data availability and quality, and interpretability.

Longitudinal patient-level data on hospital admissions

We used the Hospital Episode Statistics Admitted Patient Care (HES APC) database to derive our exposure of interest and other maternal and child characteristics near to birth (Table 1). HES APC captures all National Health Service funded hospital admissions in England and covers 97% of all births and 98-99% of all hospital admissions.[21] HES APC consists of records by episode of inpatient care, each with a pseudonymised patient identifier attached allowing researchers to longitudinally link inpatient episodes over a patient's life course. Each inpatient episode record captures up to 20 (14 before April 2007) patient diagnoses using the International Statistical Classification of Diseases and Related Health Problems 10th Revision (ICD-10).[22]

For peer review only - http://bmjopen.bmj.com/site/about/guidelines.xhtml

We created an extract that included all singleton live births recorded in HES APC between 1st April 2006 and 31st March 2014. We derived a look-back period for each birth, which included all recorded maternal inpatient episodes within the three years prior to delivery to maximise identification of risk-factors prior to birth. We excluded births where all recorded maternal areas of residence over the look-back period – identified via the lower-layer super output area (LSOA) code – were non-English or missing as we required an English LSOA to derive maternal deprivation status (via the 2010 English Index of Multiple Deprivation).[23] Using Harron et al's longitudinal linkage of HES APC for mothers and babies in England,[24] we also had access to the child's HES APC records for 96% of births in the extract.

Our exposure of interest - the proportion of live births with maternal history of adversity-related hospital admission (ARA) - was defined as any hospital admission related to substance misuse, exposure to violence, or mental health problems during the look-back period using mutually non-exclusive ICD-10 code lists (for ARA ICD-10 codes, see Appendix page 6).[25–28] We also derived four further explanatory measures: 1) the proportion of live births where maternal age was under 20 years old, 2) the proportion of live births where maternal LSOA history was within 10% most deprived LSOAs in England; 3) the proportion of live births with low birth weight, and 4) the proportion of live births where the child was diagnosed with a complex chronic condition in early childhood (Table 1).[29] For further information on this extract, see Appendix pages 4-5.

Publicly-available data

We obtained all other yearly LA figures for risk factors from publicly-available data. We used data from the Census 2011 to derive the percentage of dependent child households with a lone parent (i.e. single-parent households) and used LA-specific rates of violent crime published by Public Health England (PHE) as a proxy measure for prevalence of LA violence.[30,31] We also included LA population size in our set of explanatory measures, from the ONS mid-year population estimates,[19] to account for differences between LAs with larger and smaller resident populations.

For peer review only - http://bmjopen.bmj.com/site/about/guidelines.xhtml

Table 1: Measures used in this study

Measure type	Measure	Temporal coverage	Description	Data source(s)	Limitations
**Outcome**	rate of infant entry to care	2006/07 to 2013/14	The number of children who first enter care during infancy, per 10,000 infants in the LA population, by financial year of first entry.	CLA return (linked by LA to ONS mid-year infant population estimates)	If a child in care is transferred to the care of another LA, or is adopted but later returns to care, they will receive a new ID number. This could lead to double counting; however, LA transfers and adoption breakdowns are uncommon.
**Descriptive (i.e. not used in modelling)**	Number of singleton live births recorded in HES APC	2006/07 to 2013/14	Number of singleton live births recorded in HES APC where maternal age is non-missing and there is at least one English LSOA recorded in maternal HES APC record in the look-back period.	HES APC	We only had access to data where date of birth was non-missing, therefore births where maternal age was missing are not captured in this analysis.<br><br>Two LAs were excluded for having fewer than 100 singleton live births in at least one financial year between 2006/07 and 2013/14.
**Explanatory**	LA population size	2006/07 to 2013/14	Number of individuals living in the local authority (LA).	ONS mid-year population estimates	ONS have only provided information on the accuracy of estimates from 2013 onwards.
**Explanatory**	% of live births with maternal history of ARA	2006/07 to 2013/14	% of singleton live births recorded in hospital where mother had at least one adversity-related hospital admission (ARA) [a] in the three years prior to delivery.	HES APC	Up to 20 ICD-10 codes are available per episode of inpatient care in HES APC (up to 14 in 2006/07); however, the number of codes recorded likely differs among hospitals. This may result in underestimation of this measure in some local authorities.
**Explanatory**	% of live births where mother < 20 years old	2006/07 to 2013/14	% of singleton live births recorded in hospital where the mother was less than 20 years old at delivery.	HES APC	There were very few quality issues with birth dates in the HES APC extract (e.g. < 10 or > 50 years old at delivery).

For peer review only - http://bmjopen.bmj.com/site/about/guidelines.xhtml

**Explanatory**	% of live births where maternal LSOA history within 10% most deprived LSOAs in England	2006/07 to 2013/14	% of singleton live births recorded in hospital where mother lived in an LSOA that was one of the 10% most deprived LSOAs in England (according to the 2010 Index of Multiple Deprivation - IMD) within the three years prior to delivery.	HES APC (linked by LSOA to 2010 IMD measures)	The LSOA used to derive maternal deprivation could be up to three years out of date at time of delivery. In addition, where women with multiple LSOAs recorded in the look-back period, each LSOA was linked to the 2010 IMD deciles and the minimum decile of deprivation (i.e. most deprived) from all LSOAs recorded was selected.
**Explanatory**	% of live births where child has a complex chronic condition	2006/07 to 2013/14	% of singleton live births with mother-baby linkage where the child was diagnosed with a complex chronic condition[b] in the first 2 years of life or died before the age of 5 from a complex chronic condition- related cause.	HES APC	Information on children with complex chronic conditions was only available for births with mother-baby record linkage. Therefore this measure was calculated using only singleton live births with linkage available.<br><br>A further nine LAs were excluded as they were missing mother-baby record linkage for more than 35% of singleton live births in at least one financial year between 2006/07 and 2013/14.
**Explanatory**	% of live births with low birth weight	2010/11	% of singleton live births where child had a low birth weight - identified where recorded birthweight < 2500g or a low birth weight- related ICD-10 code (P05.0, P07.0 or P07.1) was recorded in child's HES APC record within seven days of delivery.	HES APC	There considerable variation in quality of birth weight recording by hospitals. Where birth weight was missing in the delivery record but mother-baby linkage was available, we looked for recorded birth weight in the child's birth record and for ICD-10 codes related to low birth weight. The quality of birth weight recording also varied from year to year and therefore we decided to use data only from the 2010/11 year (the mid-point of our study period).<br><br>A further nine LAs were excluded as they were missing a recorded birthweight in the maternal or child (where available) HES APC record at birth for more than 35% of singleton live births between April 2010 and March 2011.

For peer review only - http://bmjopen.bmj.com/site/about/guidelines.xhtml

**Explanatory**	% of dependent child households with lone parent	2011	% of households with dependent children (i.e. children aged 0-15 years old), where there is a single parent.	Census 2011 (Table LC1109EW)	
**Explanatory**	Rate of violent crime (per 100 LA residents)	2010/11	The number of violence against the person offences, based on police recorded crime data, per 100 people resident in the LA.	Public Health England Fingertips (Indicator 11202)	This does not capture violent crimes not reported to, or recorded by, the police. In addition, rate of violent crime in city centres with few residents (such as City of London) may be inflated as there will be large numbers of people commuting into these areas who are not counted in the population denominator.

ARA = adversity-related hospital admission; **CLA** = Children Looked After; **HES APC** = Hospital Episode Statistics Admitted Patient Care; **ICD-10** = International Statistical Classification of Diseases and Related Health Problems 10th Revision; **IMD** = Index of Multiple Deprivation; **LA** = local authority; **LSOA** = lower-layer super output area; **ONS** = Office for National Statistics.

[a] We defined history of ARA as any episode of admitted patient care related to substance misuse, mental health problems (including self-harm) or exposure to violence in the look-back period, determined by several non-mutually exclusive lists of ICD-10 codes.[25–28]
[b] Diagnoses of complex chronic conditions were identified using Feudtner et al's ICD-10 code list.[29]

For peer review only - http://bmjopen.bmj.com/site/about/guidelines.xhtml

Longitudinal modelling

We analysed the relationship over time between the LA-specific, yearly rate of infant entry into care (which were approximately normally distributed) and LA-specific, yearly percentage of singleton live-births with a maternal history of ARA, using linear mixed-effect models with restricted maximum likelihood estimation (for histograms of the outcome by financial year, see Appendix page 7). LA-specific percentage of live births with maternal history of ARA was modelled as a time-varying covariate between 2006/07 and 2013/14. The model coefficient for this covariate could be interpreted as the effect of an increase either in the same LA (within effect) or in different LAs (between effect), assuming these effects are equivalent, which may not be the case. Therefore, we allowed the between- and within- LA effects to differ from one another by replacing the original LA-specific variable with two variables: (1) the mean value over the study period, and (2) the difference from this mean for each yearly value. The coefficient for the first variable captures the between-LA effect and the coefficient for the second the within-LA effect.[32] We used Wald χ^2 tests to test the null hypothesis that these two effects were equal. All other LA-level risk factors for entry into care were included in models as non-time varying variables using data from 2010/11 only (i.e. midpoint of study period) as we observed minor variations and this improved model parsimony.

We fitted five models: (1) a ('null') model with only financial year as the explanatory variable; (2) a model with financial year and both LA-specific mean and mean-centred maternal ARA prevalence among live births; (3) a model which included all explanatory measures (as listed in Table 1), including mean and mean-centred maternal ARA prevalence; (4) a model which included all explanatory measures and the original maternal ARA prevalence; and (5) model 4 with an interaction between financial year and maternal ARA prevalence. Models (1) to (4) included random-intercepts for LA and random-slopes for financial year. Model 5 included only random-intercepts for LA as more complex random effects structures did not converge.

The assumption of normality for the level-1 residuals of each model was checked using quantile-quantile plots and histograms and we inspected fixed-effect parameter estimates and standard errors for inflated values that would be symptomatic of multi-collinearity. We used the Akaike Information Criteria (AIC) to assess relative goodness-of-fit and all five models were checked for implausible predicted values. We also performed model-based parametric bootstrap (with 10,000 simulations) to estimate the proportion of variation in the outcome explained by the whole model (i.e. by both fixed- and random-effects) and by only the fixed-effects using formulas for a conditional and marginal pseudo R^2 value, respectively.[33–35] We reported the median marginal and conditional pseudo R^2 values from the bootstrapped samples, along with 95% confidence intervals (using the percentile method). All data management and analysis were carried out using R version 3.5.1.

Role of the funding source

The funder had no role in the study design, data collection, analysis, interpretation, or writing of the report. The corresponding author had full access to all the data in the study and had final responsibility for the decision to submit for publication.

Patient and Public Involvement

No patients or members of the public were involved in the development or analyses of the current study. However, this study was carried out to inform a wider piece of work: linkage of administrative family court and healthcare data to better understand the healthcare need and health service use of

For peer review only - http://bmjopen.bmj.com/site/about/guidelines.xhtml

women involved in care proceedings in England, particularly those with a mental health illness. For this larger project, we attended two regular group meetings of mental health service users to discuss the project and the data linkage between administrative family court data and mental health service records in England. We also held a focus group with women who have been involved in care proceedings to discuss the study in more detail and gain feedback on the project objectives. These public involvement sessions have helped us to strengthen our research plan, improving the relevance of future findings to the population under study.

RESULTS

For peer review only

For peer review only - http://bmjopen.bmj.com/site/about/guidelines.xhtml

Table 2: Local authority characteristics

LA characteristics (median [min, max])	2006/07 (N = 131)	2007/08 (N = 131)	2008/09 (N = 131)	2009/10 (N = 131)	2010/11 (N = 131)	2011/12 (N = 131)	2012/13 (N = 131)	2013/14 (N = 131)
Number of singleton live births recorded in HES APC	3288 [323, 16,076]	3416 [299, 16,687]	3454 [295, 16,847]	3516 [309, 16,722]	3552 [264, 16,734]	3550 [298, 17,132]	3440 [333, 16,932]	3415 [303, 16,438]
Rate of infant entry to care (per 10,000)	72.76 [0.00, 240.00]	66.25 [8.83, 184.70]	72.99 [4.62, 197.18]	79.19 [0.00, 280.26]	81.89 [4.51, 195.74]	90.16 [10.26, 253.56]	93.05 [0.00, 318.51]	90.14 [13.94, 269.50]
LA Population size	56,455 [8000, 313,364]	56,783 [8140, 315,580]	57,453 [8114, 317,556]	58,339 [7995, 318,654]	58,570 [8079, 320,618]	59,039 [8031, 322,743]	60,307 [7809, 324,341]	60,426 [7795, 326,092]
% of live births with maternal history of ARA	2.73 [0.52, 10.07]	2.89 [0.95, 8.91]	3.15 [1.19, 9.09]	3.66 [0.90, 9.36]	4.33 [1.49, 11.14]	5.21 [1.43, 12.53]	6.15 [1.71, 15.94]	7.01 [3.12, 16.19]
% of live births where mother < 20 years old	7.01 [1.14, 14.50]	6.82 [1.46, 14.13]	6.58 [1.35, 12.82]	6.44 [1.50, 13.89]	5.72 [1.17, 11.89]	5.39 [1.13, 10.77]	5.16 [1.30, 9.93]	4.33 [0.87, 8.49]
% of live births where maternal LSOA history within 10% most deprived LSOAs in England	14.46 [0.00, 60.18]	14.98 [0.00, 60.46]	14.64 [0.00, 61.92]	15.33 [0.00, 60.90]	14.87 [0.00, 60.36]	14.75 [0.11, 61.21]	14.47 [0.00, 61.52]	14.72 [0.24, 60.68]
% of live births where child has a complex chronic condition	1.64 [0.60, 3.34]	1.64 [0.84, 3.25]	1.62 [0.60, 2.74]	1.78 [0.84, 3.68]	1.78 [0.93, 3.19]	1.81 [1.06, 3.38]	1.83 [1.11, 3.61]	1.93 [1.03, 3.22]
% of live births with low birth weight					6.26 [4.22, 9.94]			
% of dependent child households with lone parent					18.31 [9.78, 30.94]			
Rate of violent crime (per 100 LA residents)					1.14 [0.52, 3.17]			

ARA = adversity-related hospital admission; **HES APC** = Hospital Episode Statistics Admitted Patient Care; **LA** = local authority; **LSOA** = lower-layer super output area.

For peer review only - http://bmjopen.bmj.com/site/about/guidelines.xhtml

Table 2 summarises the LA characteristics for the study cohort by financial year. The median number of residents per LA increased over time (56455 residents in 2006/07 vs 60426 in 2013/14), while the median number of live births (3288 in 2006/07 vs 3415 in 2013/14) and the median percentage of live births where maternal LSOA history was within the 10% most deprived English LSOAs (14·46% in 2006/07 vs 14·72% in 2013/14) remained stable. Across all 131 LAs, the median rate of infant entry into care (72·76 per 10,000 in 2006/07 vs 90·14 in 2013/14) and the median percentage of live births with maternal history of ARA (2·73% in 2006/07 vs 7·01 in 2013/14) increased over time. LA-specific rates for both these measures varied substantially each year between LAs. The median percentage of live births where the child had a complex chronic condition in early childhood also increased over time (1·64% in 2006/07 vs 1·93% in 2013/14), though LA variation decreased over time (min-max: 0·60-3·34% in 2006/07 vs 1·03-3·22% in 2013/14). Both the median percentage of live births to mothers under 20 years old (7·01% in 2006/07 vs 4·33% in 2013/14) and the LA variation in this measure (min-max: 1·14-14·50% in 2006/07 vs 0·87-8·49% in 2013/14) decreased over time. There was variation between LAs in the proportion of live births with low birth weight (min-max: 4·22-9·94%), the rate of violent crime (0·52-3·17 per 100 residents) and the proportion of dependent child households with a lone parent (9·78-30·94%).

[Insert Figure 2 – Figure 2: Modelling the association between LA-specific percentage of live births with maternal history of ARA and LA-specific rate of infant entry into care for 131 English local authorities, over time (2006/07 to 2013/14)]

Figure 2 footnotes:

* Models were adjusted for all other explanatory measures (Table 1).

Notes: the term 'fixed-effects' includes any explanatory measures in the model such as time and maternal history of ARA but does not include random-effects such as random intercepts and random slopes; 95% confidence intervals given in brackets.

[a] Effect where the percentage of live births with maternal history of ARA equal to zero.

[b] Effect in 2006/07.

Modelling the association between rate of infant entry into care and prevalence of maternal adversity-related hospital admissions before birth

Figure 2A displays point estimates and 95% confidence intervals from Models (1) to (5) for the coefficients of the time-varying covariates. Prior to adjustment (i.e. Model 2), a percentage point increase in percentage of live births with maternal history of ARA within the same LA was associated with an extra 2·44 infants, per 10,000, entering care (95% CI: 1·10 to 3·78), while a percentage point increase between two different LAs was associated with an extra 11·63 infants, per 10,000, entering care (8·94 to 14·31). Using Models (1) and (2), we estimated that the percentage of live births with a maternal history of ARA explained 24% (95% CI: 14-35%) of the variation in the rate of infant entry into care between 2006/07 and 2013/14 (Figure 2B). After adjustment for all other explanatory measures (i.e. Model 3), there was insufficient evidence that the effect on the outcome of increases to the percentage of live births with maternal history of ARA within the same LA and the effect on the outcome of increases between different LAs were different (p-value = 0.36). After refitting the adjusted model with the original (not disaggregated) version of this measure (i.e. Model 4), there was evidence that a 1% point increase in the percentage of live births with maternal history of ARA, either within the same LA or between two different LAs, was associated with an extra 2·56 infants

For peer review only - http://bmjopen.bmj.com/site/about/guidelines.xhtml

per 10,000 entering care (1·31 to 3·82) over a 12-month period, holding all other model covariates constant. Finally, we explored whether the effect of an increase within the same LA or between two different LAs in percentage of live births with maternal history of ARA varied over the study period (i.e. Model 5). There was evidence that the magnitude of the association between the percentage of live births with maternal history of ARA (by LA, over the study period) and rate of infant entry into care increased over time between 2006/07 and 2013/14 (interaction coefficient estimate: 0·44, 95% CI: 0·15 to 1·44), as seen in Figure 3. (For full model results, see Appendix pages 8-10).

[Insert Figure 3 here - Figure 3: Exploring changes in the association between the LA-specific percentage of live births with maternal history of ARA and LA-specific rate of infant entry into care between 2006/07 and 2013/14]

For peer review only

For peer review only - http://bmjopen.bmj.com/site/about/guidelines.xhtml

DISCUSSION

Increases either between LAs or within the same LA over time in the rate of infant entry into care were found to be associated with an increased prevalence of maternal history of adversity related hospital admission (ARA). Evidence for this association persisted even after controlling for other LA-level risk factors for entry to care. The magnitude of the increase in rate of infant entry into care per percentage point increase in the percentage of live births with maternal history of ARA increased over time, particularly from 2009/10 onwards (Figure 3). We estimated that the percentage of live births with maternal history of ARA alone explained between 14-35% of the LA variation in rate of infant entry into care over the study period. The final model, with all covariates included, explained 47-60% of this variation.

Strengths

A key strength of this study is the breadth of information on mothers and children included in the models. Six out of nine of our measures were derived using two national, longitudinal databases (HES APC and CLA), each with person-level records enabling follow up through health and social care services throughout England over time. In particular, HES APC captures diagnoses via ICD-10 codes, allowing us to identify adversity-related healthcare need among mothers up to three years before delivery (i.e. maternal history of ARA) that was sufficiently severe to be recorded during a hospital admission. Many of the ARA codes have been previously validated in other populations.[36–39] Another strength is the inclusion of multiple risk factors for maltreatment and infant entry into care in statistical modelling. We designed our modelling approach to balance model parsimony with adjustment for confounders that were supported by external evidence, which were relevant to policy and measurable. We further preserved model parsimony by allowing only our main exposure (maternal history of ARA) to vary over time, while fixing all other model covariates at their 2010/11 values. Most of the explanatory measures in this final model were derived from administrative hospital records (HES APC), highlighting the importance of considering indicators for parental health near to birth when exploring LA variation in rate of infant entry to care.

Limitations

The main limitation of this study sits with its ecological design. While we have found that increases in the percentage of live births with maternal history of ARA are associated with increases in the rate of infant entry to care over a given area, we cannot examine, at an individual level, whether children born to women with a history of adversity-related admission prior to birth were more likely to be placed into care during infancy. However, there is currently no English database containing mother-to-baby linked healthcare data with onward linkage to information on children's social care outcomes. We, therefore, studied the association between maternal adversity prior to birth and infant entry to care at an LA level. There is also a lack of information on 'supply-side' factors to infant entry to care, such as funding and availability of foster care, of sufficient quality for research. A further limitation is that we cannot distinguish whether increases over time in the percentage of live births with maternal history of ARA reflect a true increase or are partly explained by nationwide changes in coding practices, although adversity admissions appear to be increasing particularly among younger women.[40] Finally, we were limited to maternal and not paternal adversity-related hospital admission as it is not possible to identify fathers in HES APC. We were also restricted to hospital indicators of adversity as primary care indicators cannot be linked to local authority.

For peer review only - http://bmjopen.bmj.com/site/about/guidelines.xhtml

Implications of the study findings

Maternal hospital admissions related to adversity such as substance misuse, exposure to violence, and mental health problems explained a substantial proportion of LA variation in the rate of infant entry into care and the contribution of this risk factor is increasing with time. These admissions present opportunities to respond to adversity-related healthcare need prior to pregnancy and birth. However, parents and parents-to-be who are subject to children's social care involvement often have a relatively short window in which to make changes; therefore, services targeted to this population must offer timely access to ensure support is available within the timeframes of children's social care assessments and family court proceedings.

We saw increased acceleration over time in the average LA percentage of live births with maternal history of adversity-related hospital admission and increased magnitude over time in the effect of increases to LA percentage of live births with history of maternal adversity-related hospital admission on the rate of infant entry into care, which coincided with the introduction of cuts to central government funding for LAs.[41] These cuts led many LAs to decrease spend on early intervention, youth services, and some public health programmes.[42,43] However, it was beyond the scope of this work to investigate the effect of austerity on changes to the association between the percentage of live births with maternal history of ARA and rate of infant entry into care over time.

This study highlights the importance of establishing a linked parent-child healthcare data resource with onward linkage to CLA and other social care data, to enable more robust evaluation of the association between maternal ARA and other health indicators and infant entry into care to inform preventive interventions. Such data linkages would be vital to inform policy strategies aimed at improving women's health, wellbeing, and reproductive rights, and potentially reduce infant entries to care.

For peer review only

For peer review only - http://bmjopen.bmj.com/site/about/guidelines.xhtml

REFERENCES

1 Department for Education. Statistics: looked-after children. 2019. https://www.gov.uk/government/collections/statistics-looked-after-children (accessed Sept 26, 2019).

2 Family Rights Group. Care Crisis Review: options for change. 2018. https://www.frg.org.uk/images/Care_Crisis/CCR-FINAL.pdf (accessed Oct 1, 2019).

3 Oranga Tamariki. Babies and children entering Oranga Tamariki care. 2019. https://www.orangatamariki.govt.nz/assets/Uploads/OIA-responses/children-in-care/20190628-Babies-and-children-entering-Oranga-Tamariki-care.pdf (accessed Oct 17, 2019).

4 Woods R, Henderson G. Changes in out of home care and permanence planning among young children in Scotland, 2003 to 2017. *Adopt Foster* 2018. DOI:10.1177/0308575918790435.

5 Marsh CA, Browne J, Taylor J, Davis D. Characteristics and outcomes of newborns entered who entered into care (EIC) within 7 days of birth in NSW, Australia. *Child Youth Serv Rev* 2017. DOI:10.1016/j.childyouth.2017.08.005.

6 Australian Institute of Health and Welfare. Data tables: Child protection Australia 2017-18. 2019. https://www.aihw.gov.au/reports/child-protection/child-protection-australia-2017-18/data (accessed Oct 17, 2019).

7 Children's Bureau. Adoption & Foster Care Statistics. https://www.acf.hhs.gov/cb/research-data-technology/statistics-research/afcars (accessed Oct 22, 2019).

8 Ghertner R, Waters A, Radel L, Crouse G. The role of substance use in child welfare caseloads. *Child Youth Serv Rev* 2018; **90**: 83–93. DOI:10.1016/j.childyouth.2018.05.015.

9 Russell J, Macgill S. Demographics, policy, and foster care rates; A Predictive Analytics Approach. *Child Youth Serv Rev* 2015; **58**: 118–26. DOI:10.1016/j.childyouth.2015.09.009.

10 Naughton AM, Maguire SA, Mann MK, *et al.* Emotional, behavioral, and developmental features indicative of neglect or emotional abuse in preschool children a systematic review. JAMA Pediatr. 2013. DOI:10.1001/jamapediatrics.2013.192.

11 Hughes K, Bellis MA, Hardcastle KA, *et al.* The effect of multiple adverse childhood experiences on health: a systematic review and meta-analysis. *Lancet Public Heal* 2017. DOI:10.1016/S2468-2667(17)30118-4.

12 Children Act 1989, s.17. England & Wales, 1989 http://www.legislation.gov.uk/ukpga/1989/41/section/17.

13 Stith SM, Liu T, Davies LC, *et al.* Risk factors in child maltreatment: A meta-analytic review of the literature. Aggress. Violent Behav. 2009. DOI:10.1016/j.avb.2006.03.006.

14 Mulder TM, Kuiper KC, van der Put CE, Stams G-JJM, Assink M. Risk factors for child neglect: A meta-analytic review. *Child Abuse Negl* 2018; **77**: 198–210. https://doi.org/10.1016/j.chiabu.2018.01.006doi.

15 Simkiss DE, Stallard N, Thorogood M. A systematic literature review of the risk factors associated with children entering public care. *Child Care Health Dev* 2013; **39**: 628–42. DOI:10.1111/cch.12010.

16 National Audit Office. Pressures on children's social care. 2019.

For peer review only - http://bmjopen.bmj.com/site/about/guidelines.xhtml

https://www.nao.org.uk/report/pressures-on-childrens-social-care/ (accessed April 10, 2019).

17 Guttmann A, Blackburn R, Amartey A, *et al.* Long-term mortality in mothers of infants with neonatal abstinence syndrome: A population-based parallel-cohort study in England and Ontario, Canada. *PLOS Med* 2019. DOI:https://doi.org/10.1371/journal.pmed.1002974.

18 O'Donnell M, Maclean M, Sims S, Brownell M, Ekuma O, Gilbert R. Entering out-of-home care during childhood: Cumulative incidence study in Canada and Australia. *Child Abus Negl* 2016. DOI:10.1016/j.chiabu.2016.07.011.

19 Office for National Statistics. Estimates of the population for the UK, England and Wales, Scotland and Northern Ireland: mid-2001 to mid-2017 detailed time series. 2018. https://www.ons.gov.uk/peoplepopulationandcommunity/populationandmigration/populationestimates/datasets/populationestimatesforukenglandandwalesscotlandandnorthernireland (accessed Sept 21, 2018).

20 Mc Grath-Lone L, Harron K, Dearden L, Nasim B, Gilbert R. Data Resource Profile: Children Looked After Return (CLA). *Int J Epidemiol* 2016; **45**: 716–717f. DOI:10.1093/ije/dyw117.

21 Herbert A, Wijlaars L, Zylbersztejn A, Cromwell D, Hardelid P. Data Resource Profile: Hospital Episode Statistics Admitted Patient Care (HES APC). *Int J Epidemiol* 2017; **46**: 1093–1093i. DOI:10.1093/ije/dyx015.

22 World Health Organization. International Statistical Classification of Diseases and Related Health Problems - 10th Revision. Geneva, 2011.

23 English indices of deprivation 2010: all domains, all sub domains and supplementary indices. 2011. https://www.gov.uk/government/statistics/english-indices-of-deprivation-2010 (accessed April 29, 2019).

24 Harron K, Gilbert R, Cromwell D, van der Meulen J. Linking Data for Mothers and Babies in De-Identified Electronic Health Data. *PLoS One* 2016; **11**: e0164667. DOI:10.1371/journal.pone.0164667.

25 Nilsson SF, Laursen TM, Hjorthøj C, Thorup A, Nordentoft M. Risk of psychiatric disorders in offspring of parents with a history of homelessness during childhood and adolescence in Denmark: a nationwide, register-based, cohort study. *Lancet Public Heal* 2017; **2**: E541–50. DOI:10.1016/S2468-2667(17)30210-4.

26 Herbert A, Gilbert R, González-Izquierdo A, Li L. Violence, self-harm and drug or alcohol misuse in adolescents admitted to hospitals in england for injury: A retrospective cohort study. *BMJ Open* 2015; **5**. DOI:10.1136/bmjopen-2014-006079.

27 Fone D, Morgan J, Fry R, *et al.* Change in alcohol outlet density and alcohol-related harm to population health (CHALICE): a comprehensive record-linked database study in Wales. *Public Heal Res* 2016. DOI:10.3310/phr04030.

28 González-Izquierdo A, Woodman J, Copley L, *et al.* Variation in recording of child maltreatment in administrative records of hospital admissions for injury in England, 1997-2009. *Arch Dis Child* 2010. DOI:10.1136/adc.2009.180216.

29 Feudtner C, Feinstein JA, Zhong W, Hall M, Dai D. Pediatric complex chronic conditions classification system version 2: Updated for ICD-10 and complex medical technology dependence and transplantation. *BMC Pediatr* 2014; **14**. DOI:10.1186/1471-2431-14-199.

30 Public Health England. Public Health England Fingertips Tool. https://fingertips.phe.org.uk/

For peer review only - http://bmjopen.bmj.com/site/about/guidelines.xhtml

(accessed July 25, 2019).

31 Office for National Statistics. 2011 Census: Local Characteristics data tables. 2011. https://www.nomisweb.co.uk/census/2011/local_characteristics (accessed March 12, 2019).

32 Rabe-Hesketh S, Skrondal A. Multilevel and Longitudinal Modeling Using Stata, 3rd edn. College Station, Texas: Stata Press, 2012.

33 Nakagawa S, Schielzeth H. A general and simple method for obtaining R2 from generalized linear mixed-effects models. *Methods Ecol Evol* 2013. DOI:10.1111/j.2041-210x.2012.00261.x.

34 Johnson PCD. Extension of Nakagawa & Schielzeth's R2GLMM to random slopes models. *Methods Ecol Evol* 2014. DOI:10.1111/2041-210X.12225.

35 Bartoń K. Package 'MuMIn': Multi-Model Inference. *Cran-R* 2018. www.cran.r-project.org/web/packages.

36 Kim H, Smith EG, Stano CM, *et al.* Validation of key behaviourally based mental health diagnoses in administrative data: Suicide attempt, alcohol abuse, illicit drug abuse and tobacco use. *BMC Health Serv Res* 2012. DOI:10.1186/1472-6963-12-18.

37 Davis KAS, Sudlow CLM, Hotopf M. Can mental health diagnoses in administrative data be used for research? A systematic review of the accuracy of routinely collected diagnoses. *BMC Psychiatry* 2016. DOI:10.1186/s12888-016-0963-x.

38 Swain RS, Taylor LG, Braver ER, Liu W, Pinheiro SP, Mosholder AD. A systematic review of validated suicide outcome classification in observational studies. *Int J Epidemiol* 2019. DOI:10.1093/ije/dyz038.

39 Gonzalez-Izquierdo A, Ward A, O'Donnell M, *et al.* Cross-country comparison of victimisation-related injury admission in children and adolescents in England and Western Australia. *BMC Health Serv Res* 2013. DOI:10.1186/1472-6963-13-260.

40 Blackburn RM, Herbert A, Wijlaars L, Gilbert R. Trends in Hospital Admissions for Nonfatal Adversity-Related Injury among Youths in England, 2002-2016. JAMA Pediatr. 2018. DOI:10.1001/jamapediatrics.2018.2516.

41 National Audit Office. Financial sustainability of local authorities 2018. 2018. https://www.nao.org.uk/wp-content/uploads/2018/03/Financial-sustainabilty-of-local-authorites-2018.pdf (accessed Oct 22, 2019).

42 British Medical Association. Feeling the squeeze. The local impact of cuts to public health budgets in England. 2018. https://www.bma.org.uk/collective-voice/policy-and-research/public-and-population-health/public-health-budgets (accessed Oct 22, 2019).

43 Kelly E, Lee T, Sibieta L, Waters T. Public Spending on Children in England: 2000 to 2020. 2018. https://www.childrenscommissioner.gov.uk/wp-content/uploads/2018/06/Public-Spending-on-Children-in-England-CCO-JUNE-2018.pdf (accessed Oct 1, 2019).

For peer review only - http://bmjopen.bmj.com/site/about/guidelines.xhtml

COMPETING INTERESTS

We declare no competing interests.

FUNDING

Linda Wijlaars, Ruth Gilbert, Rachel J Pearson and Stuart Bedston are supported by funding from the Nuffield Foundation, grant reference number KID/42838. Ruth Gilbert also receives funding from Health Data Research UK. Matthew A Jay is supported by funding from the Medical Research Council through the UCL Birkbeck Doctoral Training Partnership (MR/R502248/1). This work is supported by the NIHR GOSH BRC. This research benefits from and contributes to the NIHR Children and Families Policy Research Unit, but was not commissioned by the National Institute for Health Research (NIHR) Policy Research Programme. The views expressed are those of the author(s) and not necessarily those of the Nuffield Foundation, the NHS, the NIHR or the Department of Health.

ETHICAL APPROVAL

Both HES APC and CLA data were pseudonymised before we received them and all other data were already publicly available and anonymised, therefore this study did not require ethical approval.

ACKNOWLEDGMENTS

We would like to thank Dr Ania Zylbersztejn and Dr Katie Harron for their past work on an extract of Hospital Episode Statistics Admitted Patient Care to identify chronic disabling conditions among children and to link mother and child records, respectively. This work uses data provided by individuals and collected by local authorities, the Department for Education and the NHS as part of their care and support.

AUTHOR CONTRIBUTIONS

All authors contributed to the formulation of the research question and to the study design. LW and MAJ provided expertise on the HES APC and CLA data extracts, respectively. MAJ also reviewed content making reference to family law. SS reviewed the literature for validated ICD-10 codes to identify adversity-related healthcare use. BDS provided statistical guidance. RP carried out all data analysis and produced the first draft of this manuscript. All authors provided critical revision to the manuscript and approved the final version to publish.

DATA SHARING STATEMENT

No additional data on HES APC or CLA are available. Links to the 2011 Census and PHE Fingertips data used in this analysis are provided in Table 1. ONS population estimates for English local authorities can be found at https://www.ons.gov.uk/peoplepopulationandcommunity/populationandmigration/populationestimates.

For peer review only - http://bmjopen.bmj.com/site/about/guidelines.xhtml

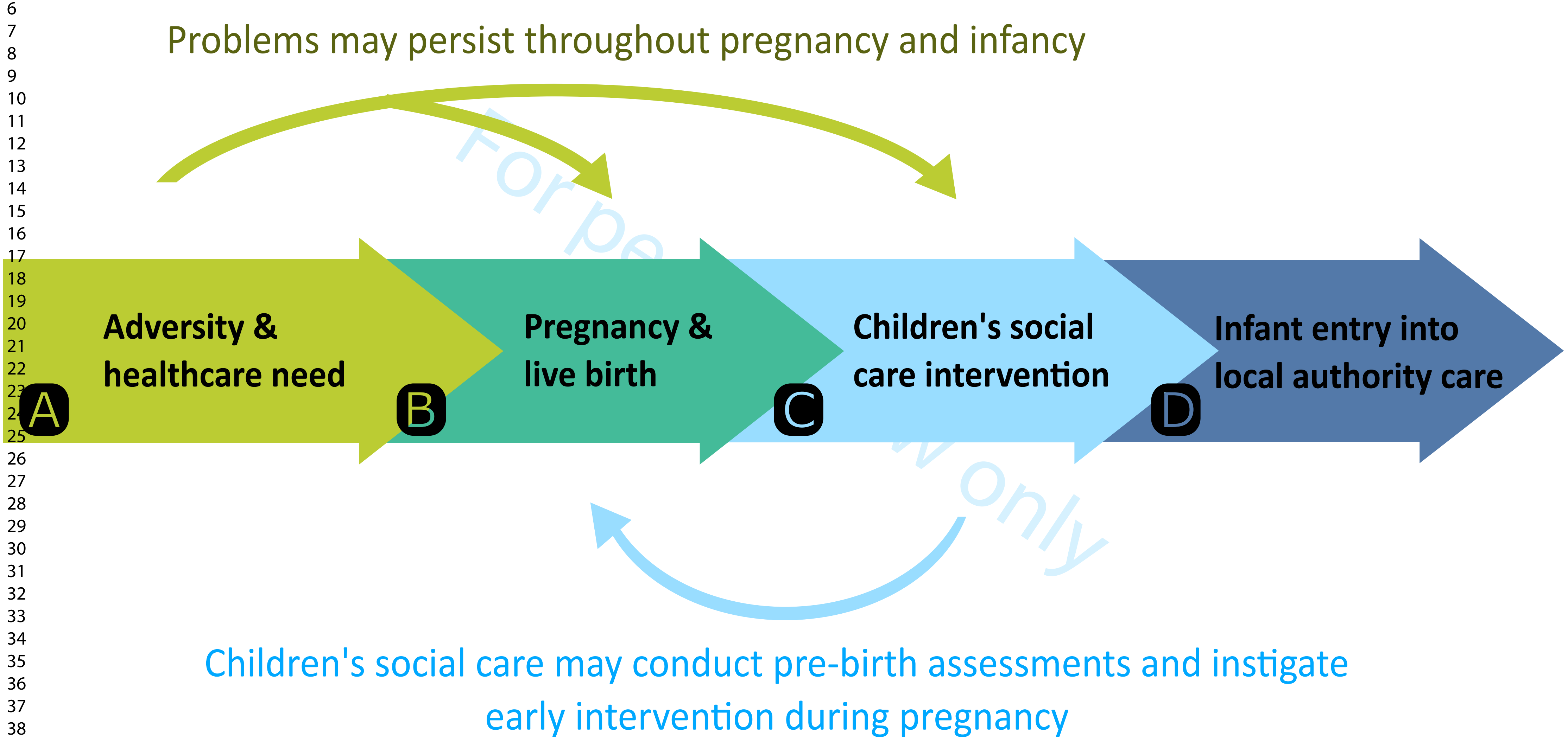
Problems may persist throughout pregnancy and infancy
A
Adversity & healthcare need
B
Pregnancy & live birth
C
Children's social care intervention
D
Infant entry into local authority care
Children's social care may conduct pre-birth assessments and instigate early intervention during pregnancy

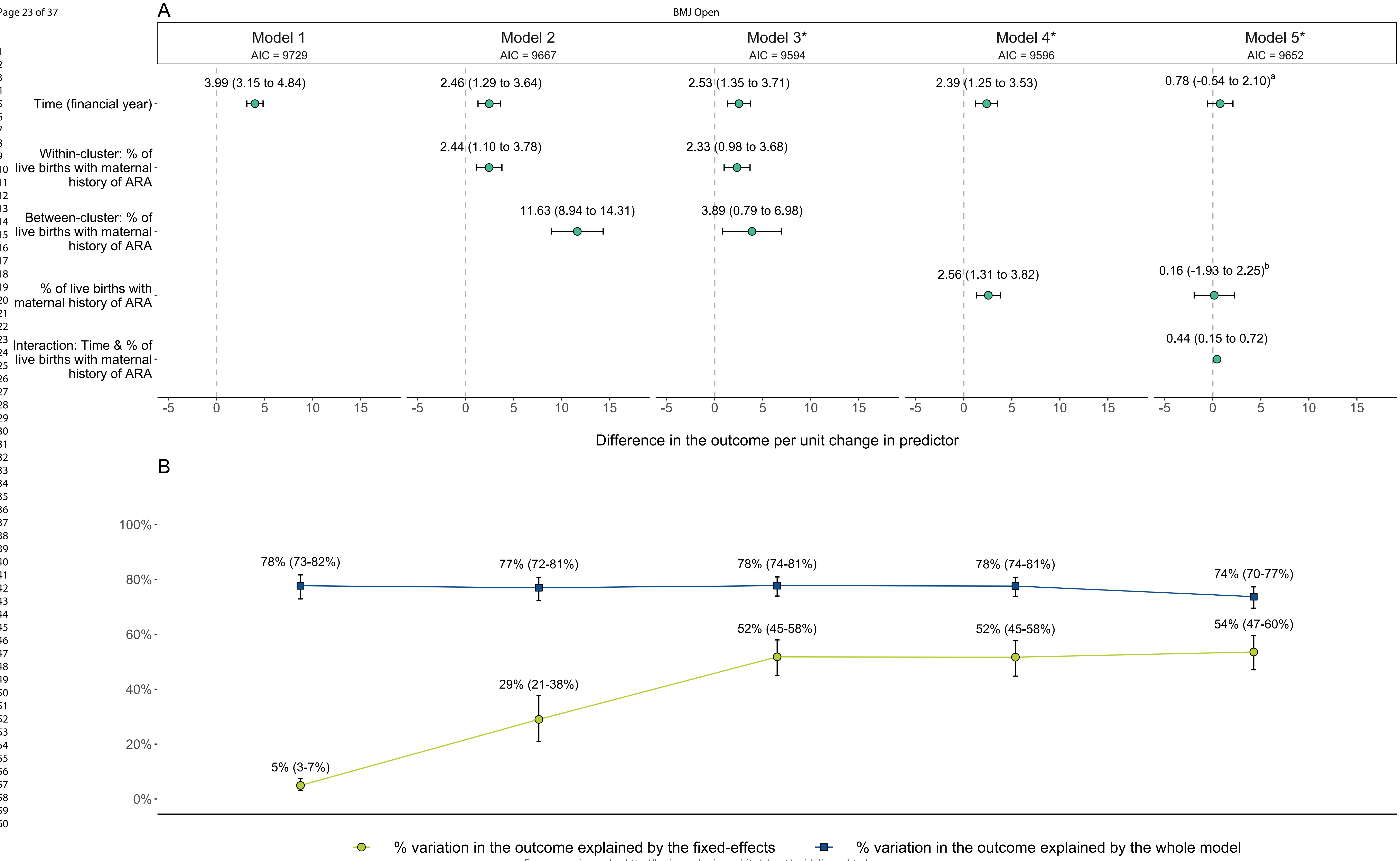
A
Model 1
AIC = 9729
Model 2
AIC = 9667
Model 3*
AIC = 9594
Model 4*
AIC = 9596
Model 5*
AIC = 9652
Time (financial year)
Within-cluster: % of live births with maternal history of ARA
Between-cluster: % of live births with maternal history of ARA
% of live births with maternal history of ARA
Interaction: Time & % of live births with maternal history of ARA
3.99 (3.15 to 4.84)
2.46 (1.29 to 3.64)
2.53 (1.35 to 3.71)
2.39 (1.25 to 3.53)
0.78 (-0.54 to 2.10)a
2.44 (1.10 to 3.78)
2.33 (0.98 to 3.68)
11.63 (8.94 to 14.31)
3.89 (0.79 to 6.98)
2.56 (1.31 to 3.82)
0.16 (-1.93 to 2.25)b
0.44 (0.15 to 0.72)
-5
0
5
10
15
Difference in the outcome per unit change in predictor
B
100%
80%
60%
40%
20%
0%
78% (73-82%)
77% (72-81%)
78% (74-81%)
78% (74-81%)
74% (70-77%)
5% (3-7%)
29% (21-38%)
52% (45-58%)
52% (45-58%)
54% (47-60%)
% variation in the outcome explained by the fixed-effects
% variation in the outcome explained by the whole model

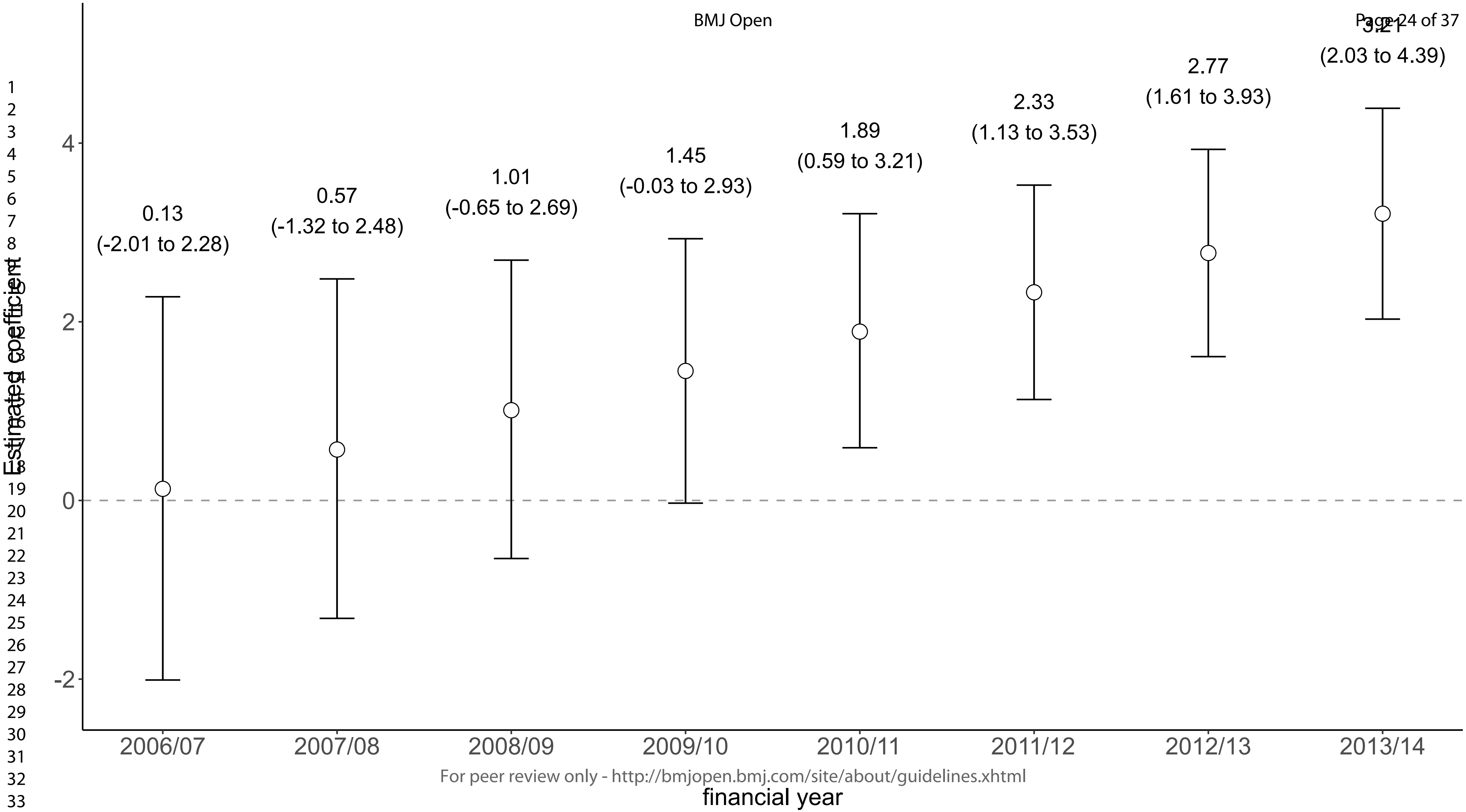
0.13
(-2.01 to 2.28)
0.57
(-1.32 to 2.48)
1.01
(-0.65 to 2.69)
1.45
(-0.03 to 2.93)
1.89
(0.59 to 3.21)
2.33
(1.13 to 3.53)
2.77
(1.61 to 3.93)
3.21
(2.03 to 4.39)
Estimated coefficient
4
2
0
-2
2006/07
2007/08
2008/09
2009/10
2010/11
2011/12
2012/13
2013/14
financial year

Supplementary appendix

Contents

Figure A1: An ecological framework of child maltreatment 2

Figure A2: A flow diagram of the Children Looked After (CLA) cohort selection 3

Missing data: CLA return 3

Figure A3: A flow diagram of the Hospital Episode Statistics Admitted Patient Care (HES APC) cohort selection 4

Missing data: HES APC 4

Table A1: Censored values in measures derived from HES APC 5

Table A2: ICD-10 code lists to identify an adversity-related hospital admission (ARA) 6

Figure A4: The distribution of rate of infant entry to care over the study period 7

Table A3 - Linear mixed-effect modelling results (Models 1-4) 8

Table A4 – Linear mixed-effect modelling results (Model 5) 10

Table A5: Conditional coefficient estimates for percentage of live births with maternal history of adversity-related hospital admissions, by financial year (Model 5) 11

For peer review only

For peer review only - http://bmjopen.bmj.com/site/about/guidelines.xhtml

Figure A1: An ecological framework of child maltreatment

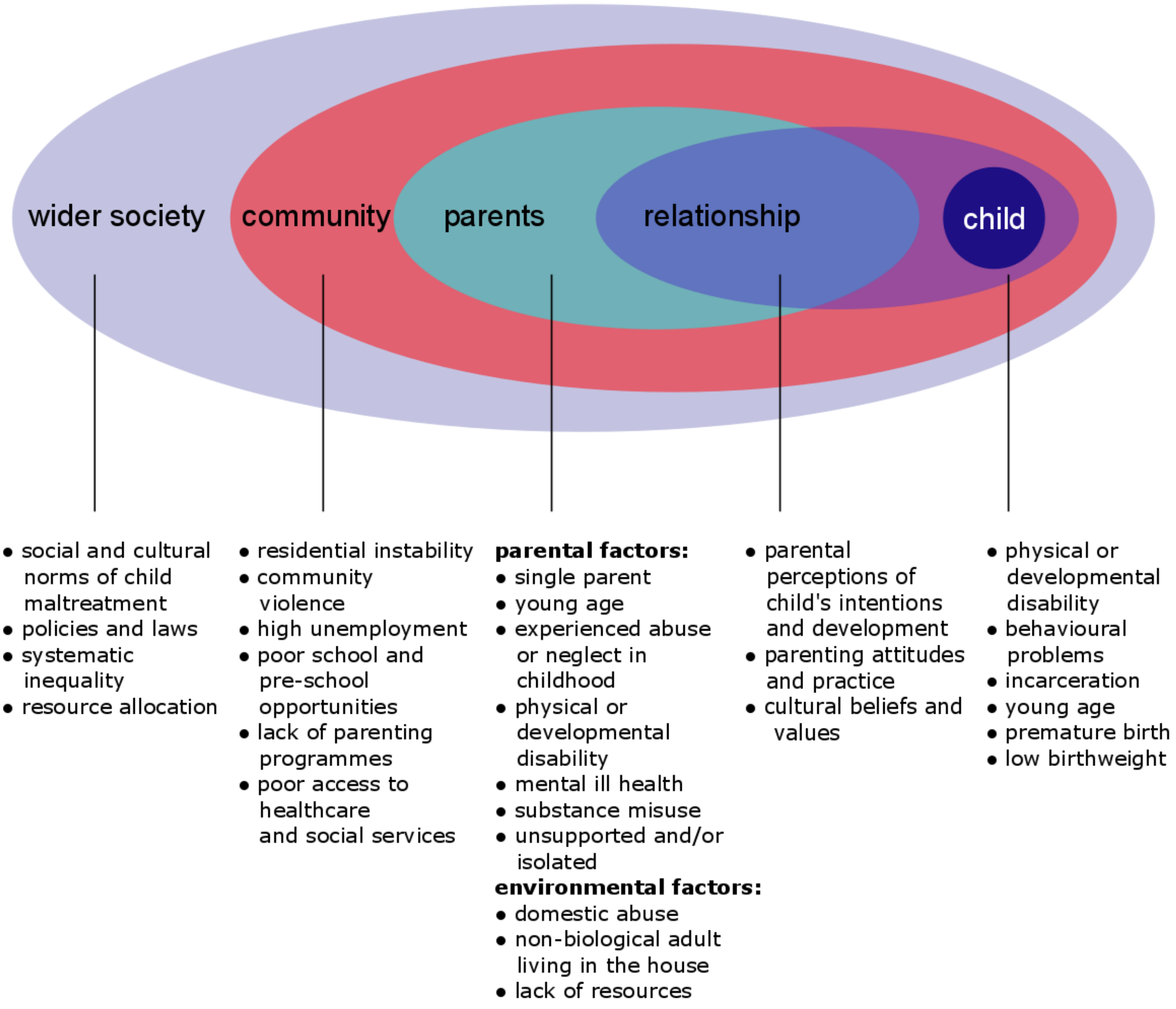

Recognising the ecological and interconnected nature of risk-factors for child maltreatment at different ecological levels, many have applied an ecological framework to this field of study over the years.[1–3] The ecological framework below builds upon previous diagrams and incorporates evidence from several systematic reviews looking at risk-factors for child maltreatment. [4–6]

For peer review only - http://bmjopen.bmj.com/site/about/guidelines.xhtml

Figure A2: A flow diagram of the Children Looked After (CLA) cohort selection

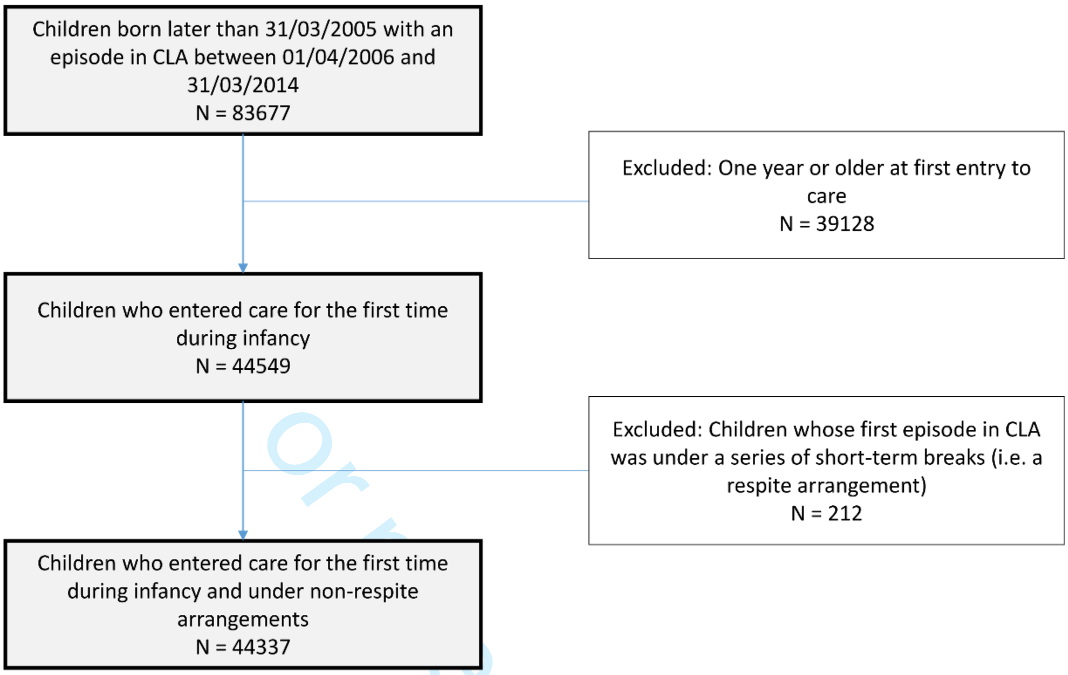

Missing data: CLA return

Adhering to the Department for Education's statistical disclosure rules, we censored any counts less than 10 before outputting them from our secure data environment. Of our 1048 LA counts of infants entering care, 60 (5.7%) had to be censored before we could output them from UCL's secure data environment. We imputed these censored values with a randomly selected number from 0 to 9 before analysis (random seed set to 1234).

For peer review only - http://bmjopen.bmj.com/site/about/guidelines.xhtml

Figure A3: A flow diagram of the Hospital Episode Statistics Admitted Patient Care (HES APC) cohort selection

We derived two cohorts using HES APC (please note that the extract of HES APC used only included individuals with dates of birth available):

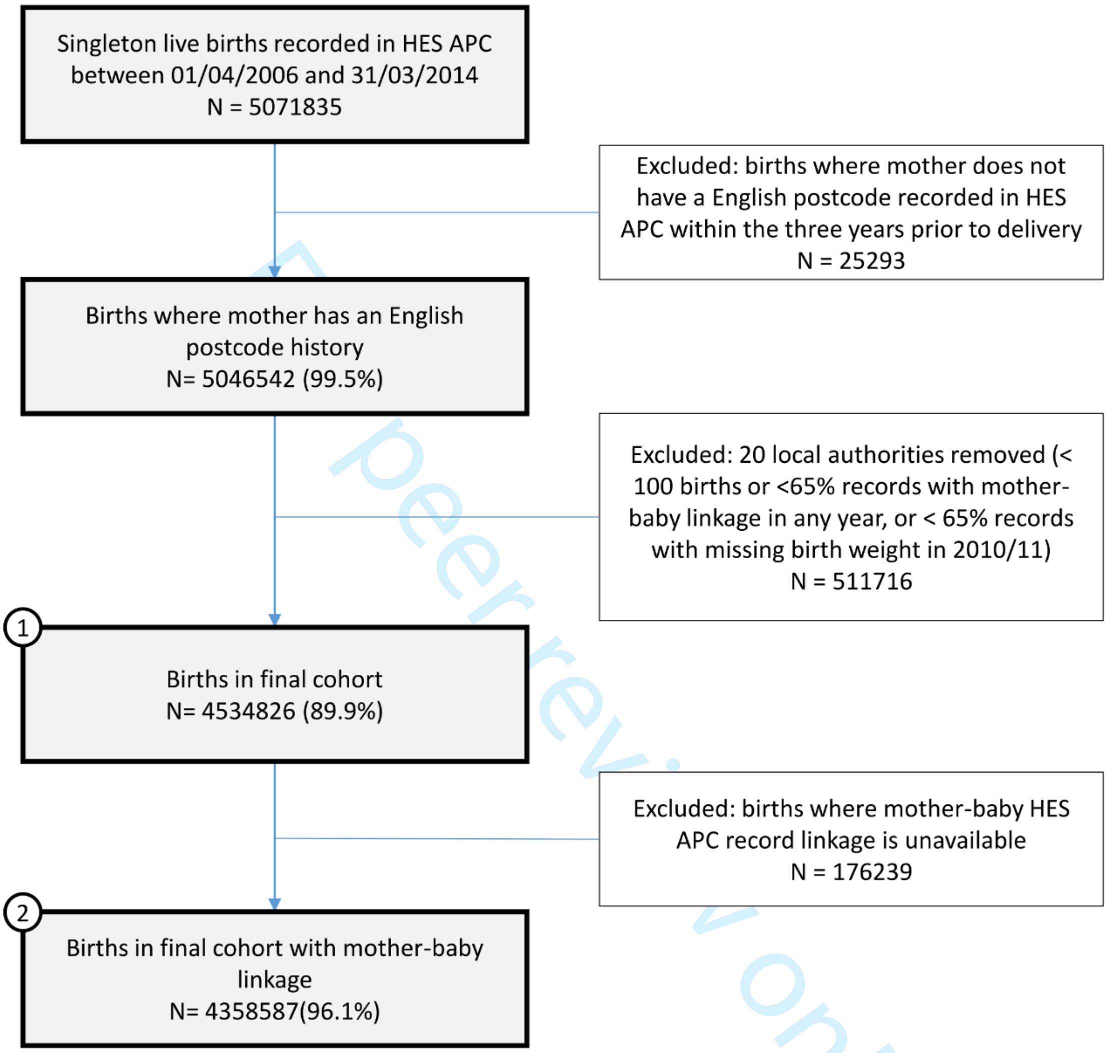

Missing data: HES APC

Adhering to the Department for Health and Social Care's statistical disclosure rules, we censored any values derived from HES APC where the underlying counts were less than five before outputting them from our secure data environment. After outputting, we imputed censored counts with a randomly selected number from 0 to 4 (random seed set to 1234).

For peer review only - http://bmjopen.bmj.com/site/about/guidelines.xhtml

Table A1: Censored values in measures derived from HES APC

Explanatory measure	Number of values	Number of values less than five (%)
% of live births with maternal history of ARA	1048	0
% of live births where mother < 20 years old	1048	0
% of live births where maternal LSOA history within 10% most deprived LSOAs in England	1048	24 (2.29%)
% of live births where child has a complex chronic condition	1048	1 (0.10%)
% of live births with low birth weight	1048	0

For peer review only

For peer review only - http://bmjopen.bmj.com/site/about/guidelines.xhtml

Table A2: ICD-10 code lists to identify an adversity-related hospital admission (ARA)

ICD-10 codes	Source
Mental Health disorders:<br>Behavioural and emotional disorder<br>F90-F95, F98<br><br>Neurotic, stress-related, and somatoform disorder<br>F40-F45, F48<br><br>Schizophrenia , Personality disorder, or Bipolar disorder<br>F20, F30-F33, F60-F62<br><br>Depressive episode or disorder<br>F32-F33<br><br>Substance use disorder<br>F10-F16, F18-F19<br><br>Other mental health disorder<br>F00-F09, F21-F25, F28, F34, F38-F39, F50-F55, F59, F63-F66, F68-F73, F79-F84, F88-F89, F99<br><br>Intentional self-harm<br>X60-X84<br><br>History of self-harm<br>Z91.5 (we also included Y87.0 – sequelae of intentional self-harm)	 <br>Nilsson, 2017<br><br><br><br><br><br><br><br><br><br><br><br><br><br><br><br><br><br><br><br>Herbert, 2015
Substance misuse:<br>Alcohol misuse<br>E51.2, T51.0, X45.0-X45.2, X45.4, X45.6, X45.8, X45.9, X65.0-X65.2, X65.4, X65.5, X65.6, X65.8, X65.9, Y15.0, Y15.2, Y15.4, Y15.8, Y15.9, Y90.0-Y91.3, Y91.9, Z50.2, Z71.4, Z72.1<br><br>Alcohol and other substance misuse<br>E24.4, F10-F16, F18-F19, G31.2, G62.1, G72.1, I42.6, K29.2, K70.0-K70.9, K85.2, K86.0, O35.4, R78.0-R78.5, T36-T50 (we did not exclude T50.6, however), X40-X44, X46-X49, X69, Y10-Y14, Y16-Y19, Z04.0, Z50.3, Z71.5, Z72.2, T51	 Fone, 2016<br><br><br><br><br>Herbert, 2015
Exposure to violence:<br>Assault<br>X85-X99, Y01-Y05, Y08-Y09<br><br>Maltreatment<br>T73-T74, Y06-Y07<br><br>Injury: undetermined cause<br>Y20-Y34, Z04.5 (we excluded Z04.8)	 Herbert, 2015

For peer review only - http://bmjopen.bmj.com/site/about/guidelines.xhtml

Figure A4: The distribution of rate of infant entry to care over the study period

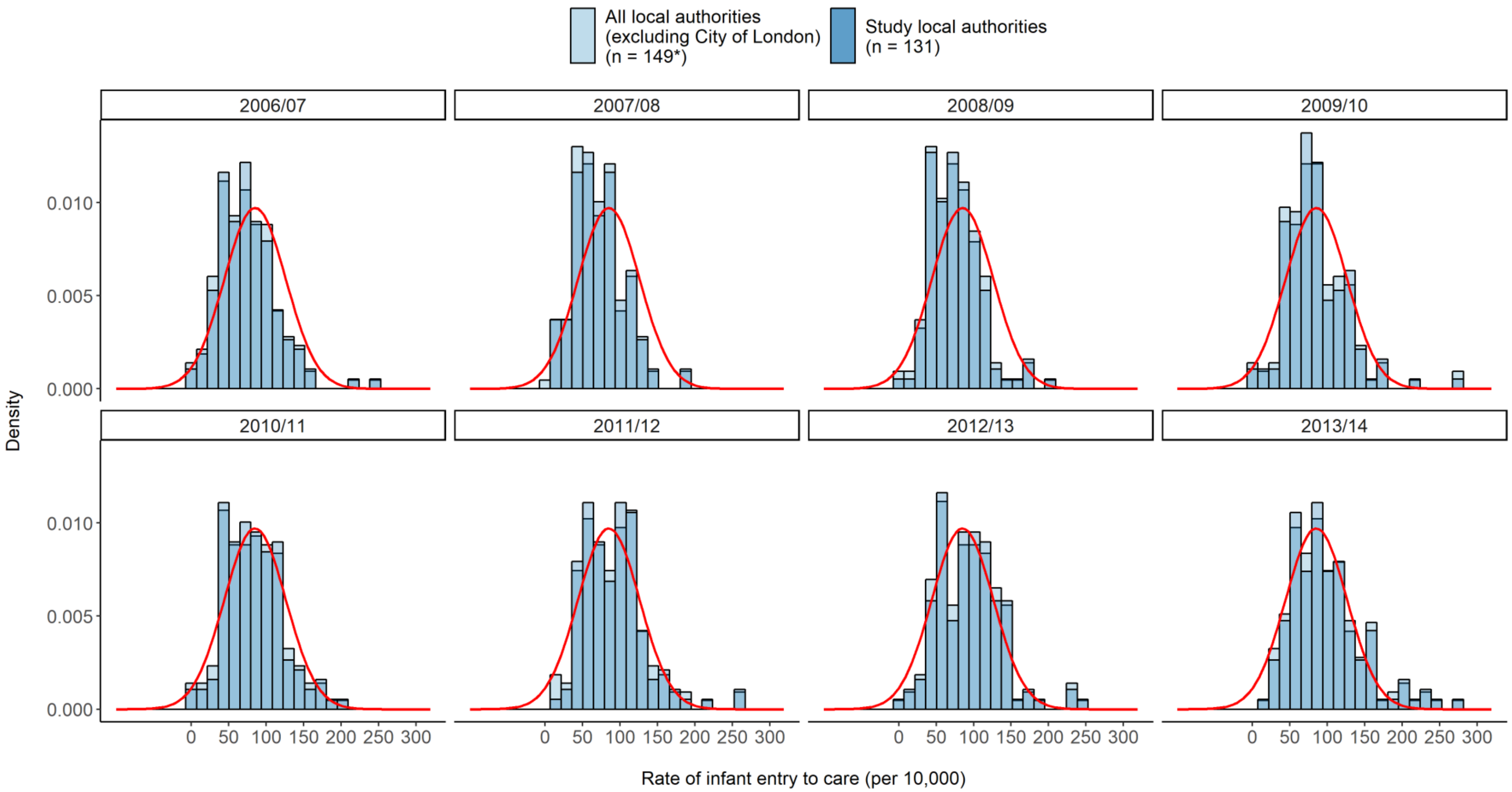

* There were 151 local authorities in 2006/07; however, the Isles of Scilly did not have any children in their care over the study period.
Data source: Children Looked After return, Department for Education.

Table A3 - Linear mixed-effect modelling results (Models 1-4)

Model parameter	Model 1		Model 2		Model 3		Model 4		Type of model parameter
	Estimate	95% C.I.	Estimate	95% C.I.	Estimate	95% C.I.	Estimate	95% C.I.	
Time (e.g. financial year Apr-March)	3.99	3.15 to 4.84	2.46	1.29 to 3.64	2.53	1.35 to 3.71	2.39	1.25 to 3.53	fixed-effect
% of live births with maternal history of ARA			(within-LA)<br>2.44<br>(between-LA)<br>11.63	1.10 to 3.78<br><br>8.94 to 14.31	2.33<br><br>3.89	0.98 to 3.68<br><br>0.79 to 6.98	(overall)<br>2.56	1.31 to 3.82	fixed-effect
% of live births where mother < 20 years old					6.77	4.50 to 9.03	7.25	5.22 to 9.28	fixed-effect
% of live births where maternal postcode history within 10% most deprived LSOAs in England					0.01	-0.35 to 0.37	0.04	-0.31 to 0.39	fixed-effect
% of live births where child has a complex chronic condition					13.42	4.55 to 22.30	14.22	5.51 to 22.93	fixed-effect
% of live births with low birth weight					1.28	-2.93 to 5.48	1.00	-3.16 to 5.17	fixed-effect
LA population size (000s)					-0.08	-0.14 to -0.02	-0.08	-0.14 to -0.02	fixed-effect
% of dependent child households with lone parent					1.87	0.65 to 3.09	1.96	0.76 to 3.17	fixed-effect
Rate of violent crime (per 100 LA residents)					6.10	-3.12 to 15.32	5.05	-3.88 to 13.98	fixed-effect
LA – random intercept (sd)	29.25		24.50		20.56		20.58		random-effect

For peer review only

Time (financial year) – random slope (sd)	3.91		3.78		3.78		3.78		random-effect
Correlation between random intercept and random slope	0.14		-0.11		-0.45		-0.45		random-effect
Residual error	19.43		19.37		19.37		19.37		residual

ARA = adversity-related hospital admission; **LA** = local authority; **LSOA** = lower-layer super output area; **sd** = standard deviation.

For peer review only

For peer review only - http://bmjopen.bmj.com/site/about/guidelines.xhtml

Table A4 – Linear mixed-effect modelling results (Model 5)

Model parameter	Estimate	95% C.I.	Type of model parameter
Time (financial year) (where average maternal ARA = 0)	0.78	-0.54 to 2.10	fixed-effect
% of live births with maternal history of ARA (in 2006/07)	0.16	-1.93 to 2.25	fixed-effect
Effect of unit increase in time on average % of live births with maternal history of ARA (or vice versa)	0.44	0.15 to 0.72	fixed-effect
% of live births where mother < 20 years old	8.29	6.23 to 10.35	fixed-effect
% of live births where maternal postcode history within 10% most deprived LSOAs in England	0.06	-0.30 to 0.41	fixed-effect
% of live births where child has a complex chronic condition	14.19	5.37 to 23.01	fixed-effect
% of live births with low birth weight	0.60	-3.62 to 4.82	fixed-effect
LA population size (000s)	-0.08	-0.15 to -0.02	fixed-effect
% of dependent child households with lone parent	1.92	0.70 to 3.14	fixed-effect
Rate of violent crime (per 100 LA residents)	5.20	-3.86 to 14.25	fixed-effect
Local Authority – random intercept (sd)	18.55		random-effect
Residual error	21.35		residual

ARA = adversity-related hospital admission; **LA** = local authority; **LSOA** = lower-layer super output area; **sd =** standard deviation.

For peer review only - http://bmjopen.bmj.com/site/about/guidelines.xhtml

Table A5: Conditional coefficient estimates for percentage of live births with maternal history of adversity-related hospital admissions, by financial year (Model 5)

Measure	Financial year	Conditional coefficient estimate	95% C.I.
Effect of a 1% point increase in % of live births with maternal history of ARA in…	2006/07	0.13	-2.01 to 2.28
	2007/08	0.57	-1.32 to 2.48
	2008/09	1.01	-0.65 to 2.69
	2009/10	1.45	-0.03 to 2.93
	2010/11	1.89	0.59 to 3.21
	2011/12	2.33	1.13 to 3.53
	2012/13	2.77	1.61 to 3.93
	2013/14	3.21	2.03 to 4.39

ARA = adversity-related hospital admission.
Note: Created using R package 'interplot' https://cran.r-project.org/web/packages/interplot/index.html.

For peer review only - http://bmjopen.bmj.com/site/about/guidelines.xhtml

References

1. Bronfenbrenner U. The Ecology of Human Development. The Educational Forum. 1979.

2. Belsky J. Child maltreatment: An ecological integration. Am Psychol. 1980;

3. Sidebotham P. An ecological approach to child abuse: A creative use of scientific models in research and practice. Child Abus Rev. 2001;10(2):97–112.

4. Stith SM, Liu T, Davies LC, et al. Risk factors in child maltreatment: A meta-analytic review of the literature. Aggression and Violent Behavior. 2009.

5. Mulder TM, Kuiper KC, van der Put CE, Stams G-JJM, Assink M. Risk factors for child neglect: A meta-analytic review. Child Abuse Negl. 2018;77:198–210.

6. Sidebotham P, Heron J, Team AS. Child maltreatment in the "children of the nineties": a cohort study of risk factors. Child Abuse Negl. 2006;

For peer review only

For peer review only - http://bmjopen.bmj.com/site/about/guidelines.xhtml

STROBE Statement—Checklist of items that should be included in reports of ***cohort studies***

	Item No	Recommendation	Reported on page
**Title and abstract**	1	(*a*) Indicate the study's design with a commonly used term in the title or the abstract	1
		(*b*) Provide in the abstract an informative and balanced summary of what was done and what was found	2
**Introduction**			
Background/rationale	2	Explain the scientific background and rationale for the investigation being reported	4
Objectives	3	State specific objectives, including any prespecified hypotheses	4
**Methods**			
Study design	4	Present key elements of study design early in the paper	4/5
Setting	5	Describe the setting, locations, and relevant dates, including periods of recruitment, exposure, follow-up, and data collection	5
Participants	6	(*a*) Give the eligibility criteria, and the sources and methods of selection of participants. Describe methods of follow-up	5-9
		(*b*) For matched studies, give matching criteria and number of exposed and unexposed	
Variables	7	Clearly define all outcomes, exposures, predictors, potential confounders, and effect modifiers. Give diagnostic criteria, if applicable	5-9
Data sources/ measurement	8*	For each variable of interest, give sources of data and details of methods of assessment (measurement). Describe comparability of assessment methods if there is more than one group	*5-9*
Bias	9	Describe any efforts to address potential sources of bias	5-9
Study size	10	Explain how the study size was arrived at	5-9
Quantitative variables	11	Explain how quantitative variables were handled in the analyses. If applicable, describe which groupings were chosen and why	5-10
Statistical methods	12	(*a*) Describe all statistical methods, including those used to control for confounding	10
		(*b*) Describe any methods used to examine subgroups and interactions	10
		(*c*) Explain how missing data were addressed	5-9
		(*d*) If applicable, explain how loss to follow-up was addressed	N/A
		(*e*) Describe any sensitivity analyses	N/A
**Results**			
Participants	13*	(a) Report numbers of individuals at each stage of study—eg numbers potentially eligible, examined for eligibility, confirmed eligible, included in the study, completing follow-up, and analysed	5
		(b) Give reasons for non-participation at each stage	N/A
		(c) Consider use of a flow diagram	appendix
Descriptive data	14*	(a) Give characteristics of study participants (eg demographic, clinical, social) and information on exposures and potential confounders	11-12
		(b) Indicate number of participants with missing data for each variable of interest	5-9

For peer review only - http://bmjopen.bmj.com/site/about/guidelines.xhtml

		(c) Summarise follow-up time (eg, average and total amount)	5
Outcome data	15*	Report numbers of outcome events or summary measures over time	11-12
Main results	16	(*a*) Give unadjusted estimates and, if applicable, confounder-adjusted estimates and their precision (eg, 95% confidence interval). Make clear which confounders were adjusted for and why they were included	12-13
		(*b*) Report category boundaries when continuous variables were categorized	N/A – no categorisation
		(*c*) If relevant, consider translating estimates of relative risk into absolute risk for a meaningful time period	N/A – AR reported
Other analyses	17	Report other analyses done—eg analyses of subgroups and interactions, and sensitivity analyses	13
**Discussion**			
Key results	18	Summarise key results with reference to study objectives	14
Limitations	19	Discuss limitations of the study, taking into account sources of potential bias or imprecision. Discuss both direction and magnitude of any potential bias	14
Interpretation	20	Give a cautious overall interpretation of results considering objectives, limitations, multiplicity of analyses, results from similar studies, and other relevant evidence	15
Generalisability	21	Discuss the generalisability (external validity) of the study results	14-15
**Other information**			
Funding	22	Give the source of funding and the role of the funders for the present study and, if applicable, for the original study on which the present article is based	19

*Give information separately for exposed and unexposed groups.

Note: An Explanation and Elaboration article discusses each checklist item and gives methodological background and published examples of transparent reporting. The STROBE checklist is best used in conjunction with this article (freely available on the Web sites of PLoS Medicine at http://www.plosmedicine.org/, Annals of Internal Medicine at http://www.annals.org/, and Epidemiology at http://www.epidem.com/). Information on the STROBE Initiative is available at http://www.strobe-statement.org.

For peer review only

For peer review only - http://bmjopen.bmj.com/site/about/guidelines.xhtml

BMJ Open

Association between health indicators of maternal adversity and the rate of infant entry to local authority care in England: a longitudinal ecological study

Journal:	*BMJ Open*
Manuscript ID	bmjopen-2019-036564.R1
Article Type:	Original research
Date Submitted by the Author:	20-Apr-2020

Complete List of Authors:	Pearson, Rachel; UCL Great Ormond Street Institute of Child Health, Population, Policy and Practice Research and Teaching Department<br>Jay, Matthew; UCL Great Ormond Street Institute of Child Health, Population, Policy and Practice Research and Teaching Department<br>Wijlaars, Linda; UCL Great Ormond Street Institute of Child Health, Population, Policy and Practice Research and Teaching Department<br>De Stavola, Bianca ; UCL Great Ormond Street Institute of Child Health, Population, Policy and Practice Research and Teaching Department<br>Syed, Shabeer; UCL Great Ormond Street Institute of Child Health, Population, Policy and Practice Research and Teaching Department<br>Bedston, Stuart; Lancaster University, Department for Sociology, Centre for Child and Family Justice Research<br>Gilbert, Ruth; UCL Great Ormond Street Institute of Child Health, Population, Policy and Practice Research and Teaching Department

**Primary Subject Heading**:	Public health
Secondary Subject Heading:	Epidemiology
Keywords:	PUBLIC HEALTH, EPIDEMIOLOGY, Child protection < PAEDIATRICS

SCHOLARONE™
Manuscripts

I, the Submitting Author has the right to grant and does grant on behalf of all authors of the Work (as defined in the below author licence), an exclusive licence and/or a non-exclusive licence for contributions from authors who are: i) UK Crown employees; ii) where BMJ has agreed a CC-BY licence shall apply, and/or iii) in accordance with the terms applicable for US Federal Government officers or employees acting as part of their official duties; on a worldwide, perpetual, irrevocable, royalty-free basis to BMJ Publishing Group Ltd ("BMJ") its licensees and where the relevant Journal is co-owned by BMJ to the co-owners of the Journal, to publish the Work in this journal and any other BMJ products and to exploit all rights, as set out in our licence.

The Submitting Author accepts and understands that any supply made under these terms is made by BMJ to the Submitting Author unless you are acting as an employee on behalf of your employer or a postgraduate student of an affiliated institution which is paying any applicable article publishing charge ("APC") for Open Access articles. Where the Submitting Author wishes to make the Work available on an Open Access basis (and intends to pay the relevant APC), the terms of reuse of such Open Access shall be governed by a Creative Commons licence – details of these licences and which Creative Commons licence will apply to this Work are set out in our licence referred to above.

Other than as permitted in any relevant BMJ Author's Self Archiving Policies, I confirm this Work has not been accepted for publication elsewhere, is not being considered for publication elsewhere and does not duplicate material already published. I confirm all authors consent to publication of this Work and authorise the granting of this licence.

For peer review only

Association between health indicators of maternal adversity and the rate of infant entry to local authority care in England: a longitudinal ecological study

Rachel J. Pearson, MSc [1]

Matthew A. Jay, MSc [1]

Linda Wijlaars, PhD [1]

Bianca L. De Stavola, PhD [1]

Shabeer Syed, MSc[1]

Stuart Bedston, PhD [2]

Ruth Gilbert, MD [1]

1 Population, Policy and Practice Research and Teaching Department
UCL Great Ormond Street Institute of Child Health
30 Guilford Street
University College London, London
WC1N 1EH

2 Centre for Child and Family Justice Research
Department of Sociology
Lancaster University
LA1 4YT

Corresponding author:
Rachel J. Pearson
222 Euston Road,
London,
NW1 2DA
Email: Rachel.Pearson@ucl.ac.uk
Tel: 020 3549 5994

For peer review only - http://bmjopen.bmj.com/site/about/guidelines.xhtml

ABSTRACT

Objective

Infants enter care at varying rates across local authorities (LAs) in England, but evidence is lacking on what is driving these differences. With this ecological study, we aimed to explore the extent to which adversity indicated within women's hospitalisation histories, pre-delivery, explained rate of infant entry into care.

Methods

We used two longitudinal person-level data sets on hospitalisations and entries to care to create annual measures for 131 English LAs, between 2006/07 and 2013/14 (Apr-Mar). We combined these measures by LA and financial year, along with other publicly-available data on LA characteristics. We used linear mixed-effects models to analyse the relationship between the outcome - LA-specific rate of infant entry into care (per 10,000 infants in the LA population) - and LA-specific percentage of livebirths with maternal history of adversity-related hospital admissions (i.e. substance misuse, mental health problems or violence-related admissions in the three years before delivery), adjusted for other predictors for entry into care.

Results

Rate of infant entry into care (mean: 85.16 per 10,000, SD: 41.07) and percentage of livebirths with maternal history of adversity-related hospital admissions (4.62%, 2.44%) varied greatly by LA. Prevalence of maternal adversity accounted for 24% of the variation in rate of entry (95% CI: 14-35%). After adjustment, a percentage point increase in prevalence of maternal adversity - both within and between LAs - was associated with an estimated 2.56 (per 10,000) more infants entering care (1.31-3.82).

Conclusions

Prevalence of maternal adversity before birth helped to explain variation in LA rates of infant entry into care. Preventive interventions are needed to improve maternal well-being before and during pregnancy, and potentially reduce risk of child maltreatment and, therefore, entries to care. Evidence on who to target and data to evaluate change requires linkage between parent-child healthcare data and administrative data from children's social care.

For peer review only

For peer review only - http://bmjopen.bmj.com/site/about/guidelines.xhtml

Strengths and limitations of this study

- This is the first study to examine the relationship between LA variation in rate of entry into care in England and parental health indicators that are associated with diminished parenting capacity.
- In our analysis, we used a breadth of information on mothers and children derived from longitudinal, person-level Hospital Episode Statistics (HES) records and our outcome was derived from the Children Looked After return (CLA) database containing all entries into care in England.
- Our estimates of local authority prevalence of live births with history of maternal adversity-related hospital admission (i.e substance misuse, mental health, or violence- related admissions up to 3 years before delivery) are likely to be underestimated due to poor recording, reporting or recognition of these problems.
- Due to the lack of national data linkages between mother-to-baby linked healthcare data and children's social care outcomes in England, we could not examine whether children born to women with a history of maternal adversity-related hospital admission were more likely to be placed into care during infancy.

For peer review only

For peer review only - http://bmjopen.bmj.com/site/about/guidelines.xhtml

INTRODUCTION

England has experienced unsustainable increases in the number of children in public care in recent years, leaving its children's social care sector overburdened.[1,2] One in five children entering public care are infants (aged under one year) and the rate of infant entry into care has increased by almost 20% since 2010/11.[1] Similar patterns and increases in infant entry to care are taking place in other countries including Scotland, the US, New Zealand, and parts of Australia. There is also marked regional variation in rates of entry across each of these settings,[3–7] with limited evidence on what drives differences.[8,9] The most frequently documented entry reason among infants entering care in England is abuse and neglect (i.e. child maltreatment).[1] Maltreatment in infancy is linked to poorer physical, intellectual and behavioural development in childhood and to substance misuse, risky sexual behaviour, domestic violence and abuse, self-harm, and poorer health outcomes later in life.[10,11] Developing our understanding of the drivers of geographic variation in the prevalence of maltreatment and demand for child protection intervention in infancy is, therefore, a key public health issue.

The responsibility for placing infants into care in England rests with the 152 local authorities (LAs) (i.e. local governmental bodies with responsibilities to providing community services, such as schools, housing, and social care) who have a statutory duty to safeguard children.[12] Past studies have shown that entry into care is driven by a complex ecological framework of risk factors relating to children and their families, the local community, service thresholds and capacity, and wider society.[13–15] These studies also highlight strong associations between parental adversities - such as mental health problems or deprivation - and child maltreatment or entry into care. Experiences of substance misuse, domestic violence, and mental health problems are common among parents whose children are subject to child protection in England.[16] Parents who experience these forms of adversity are also more likely to have serious and complex healthcare needs,[17] as well as being more likely to struggle to meet their child's needs and to maltreat their child (Figure 1).[13,14] In Western Australia and Manitoba, Canada, analyses of linked record-level administrative health and social care data showed that maternal mental health, substance-related or assault-related hospital admissions were associated with a higher relative risk of child entry into care.[18] There is also evidence that children whose parents experience these forms of adversity are more likely to be subject to repeated child protection intervention.[19,20] Therefore, in this study, we examined the extent to which LA-specific prevalence of health indicators for maternal adversity prior to birth explained variation in rate of infant entry into care among LAs in England. With this work, we aim to inform national and local policy strategies to mitigate long-lasting harm to children arising from serious parental adversity and diminished capacity to parent.

[Insert Figure 1 here – Figure 1: A hypothetical (simplified) pathway from parental adversity to infant entry into care]

(A) Substance misuse, domestic violence and abuse, and mental health problems not only affect day-to-day functioning but can also lead to serious and complex healthcare needs. **(B)** These forms of adversity can also affect the capacity to parent and may result in harm to the unborn child or infant. Also, pregnancy, birth and caring for an infant place additional stress on parents which can exacerbate experiences of adversity. **(C & D)** Where children are at a significant risk of harm, children's social care services have the power to apply for a court order to receive the child into care or may otherwise receive a child into care where to do so is in the child's best interests and the parents do not object.

For peer review only - http://bmjopen.bmj.com/site/about/guidelines.xhtml

As English children's social care data is not yet linked at the child level to longitudinal healthcare data, we conducted an ecological analysis. We have explored the relationship between LA-specific rates of maternal hospitalisation before birth due to substance misuse, domestic violence and abuse, and mental health problems and LA variation in the rate of infant entry into care. We also accounted for a range of other LA-level risk factors for entry into care including LA-specific prevalence of maternal deprivation, births to teenage mothers, and community violence.

METHODS

Study design

This longitudinal, ecological analysis used yearly aggregate measures between the 2006/07 and 2013/14 financial years (Apr-Mar) for 131 out of a possible 151 (as at April 2006) English LAs, derived from several data sources. We excluded 20 LAs from our analysis for having too few live births or poor data quality in at least one measure in one or more years (Table 1). All data were de-identified or already publicly available and anonymised, therefore this study did not require ethical approval.

Outcome

Our outcome was the annual incidence rate of first entry into care during infancy (i.e. under one year old) per 10,000 infants resident in the LA. The longitudinal Children Looked After (CLA) dataset, provided by the Department for Education, was used to determine the numerator for each LA, with Office for National Statistics population estimates being used to define the denominator.[21] CLA is a statutory data collection collated by the Department for Education and contains record-level information on all children in care in England, based on annual submissions from each LA. CLA includes information on child demographics (e.g. age, sex, ethnicity etc.) and episodes in care (e.g. episode dates, legal status, placement type etc.).[22] We used a longitudinal extract of CLA, which contained episode-in-care-level information for all children who first entered care during infancy between 1st April 2005 and 31st March 2014, to derive our outcome. Infants who entered care for any reason were included, with the exception of infants who first entered care under respite arrangements (i.e. an agreed series of short-term breaks, typically provided for children with complex healthcare needs). For further information on this extract, see Appendix page 3.

Explanatory measures

We derived several other explanatory measures, selected to capture further 'demand-side' risk factors (i.e. local service need) for child maltreatment and entry into care, alongside our exposure of interest (Table 1). These measures were chosen based upon evidence, [13–15] data availability and quality, and interpretability and were used to account for potential confounders in our statistical analyses.

Longitudinal patient-level data on hospital admissions

We used the Hospital Episode Statistics Admitted Patient Care (HES APC) database to derive our exposure of interest and other maternal and child characteristics near to birth (Table 1). HES APC captures all National Health Service funded hospital admissions in England and covers 97% of all births and 98-99% of all hospital admissions.[23] HES APC consists of records by episode of inpatient care, each with a pseudonymised patient identifier attached allowing researchers to longitudinally link inpatient episodes over a patient's life course. Each inpatient episode record captures up to 20

For peer review only - http://bmjopen.bmj.com/site/about/guidelines.xhtml

(14 before April 2007) patient diagnoses using the International Statistical Classification of Diseases and Related Health Problems 10th Revision (ICD-10).[24]

We created an extract that included all singleton live births recorded in HES APC between 1st April 2006 and 31st March 2014. We derived a look-back period for each birth, which included all recorded maternal inpatient episodes within the three years prior to delivery to maximise identification of risk-factors prior to birth. We excluded births where all recorded maternal areas of residence over the look-back period – identified via the lower-layer super output area (LSOA) code – were non-English or missing as we required an English LSOA to derive maternal deprivation status (via the 2010 English Index of Multiple Deprivation).[25] Using Harron et al's longitudinal linkage of HES APC for mothers and babies in England,[26] we also had access to the child's HES APC records for 96% of births in the extract.

Our exposure of interest - the proportion of live births with maternal history of adversity-related hospital admission (ARA) - was defined as any hospital admission related to substance misuse, exposure to violence, or mental health problems during the look-back period using mutually non-exclusive ICD-10 code lists (for ARA ICD-10 codes, see Appendix page 6).[27–30] We also derived four further explanatory measures: 1) the proportion of live births where maternal age was under 20 years old, 2) the proportion of live births where maternal LSOA history was within 10% most deprived LSOAs in England; 3) the proportion of live births with low birth weight, and 4) the proportion of live births where the child was diagnosed with a congenital anomaly in early childhood (Table 1).[31] For further information on this extract, see Appendix pages 4-5.

Publicly-available data

We obtained all other yearly LA figures for risk factors from publicly-available data. We used data from the Census 2011 to derive the percentage of dependent child households with a lone parent (i.e. single-parent households) and used LA-specific rates of violent crime published by Public Health England as a proxy measure for prevalence of LA violence.[32,33] We also included LA population size in our set of explanatory measures, from the Office for National Statistics mid-year population estimates,[21] to account for differences between LAs with larger and smaller resident populations.

For peer review only

For peer review only - http://bmjopen.bmj.com/site/about/guidelines.xhtml

Table 1: Measures used in this study

Measure type	Measure	Temporal coverage	Description	Data source(s)	Limitations
**Outcome**	rate of infant entry to care	2006/07 to 2013/14	The number of children who first enter care during infancy, per 10,000 infants in the LA population, by financial year of first entry.	CLA return (linked by LA to Office for National Statistics mid-year infant population estimates)	If a child in care is transferred to the care of another LA, or is adopted but later returns to care, they will receive a new ID number. This could lead to double counting; however, LA transfers and adoption breakdowns are uncommon.
**Descriptive (i.e. not used in modelling)**	Number of singleton live births recorded in HES APC	2006/07 to 2013/14	Number of singleton live births recorded in HES APC where maternal age is non-missing and there is at least one English LSOA recorded in maternal HES APC record in the look-back period.	HES APC	We only had access to data where date of birth was non-missing, therefore births where maternal age was missing are not captured in this analysis.<br><br>Two LAs were excluded for having fewer than 100 singleton live births in at least one financial year between 2006/07 and 2013/14.
**Explanatory**	LA population size	2006/07 to 2013/14	Number of individuals living in theLA.	Office for National Statistics mid-year population estimates	The Office for National Statistics only provide information on the accuracy of estimates from 2013 onwards.
**Explanatory**	% of live births with maternal history of ARA	2006/07 to 2013/14	% of singleton live births recorded in hospital where mother had at least one adversity-related hospital admission (ARA) [a] in the three years prior to delivery.	HES APC	Up to 20 ICD-10 codes are available per episode of inpatient care in HES APC (up to 14 in 2006/07); however, the number of codes recorded likely differs among hospitals. This may result in underestimation of this measure in some local authorities.
**Explanatory**	% of live births where mother < 20 years old	2006/07 to 2013/14	% of singleton live births recorded in hospital where the mother was less than 20 years old at delivery.	HES APC	There were very few quality issues with birth dates in the HES APC extract (e.g. < 10 or > 50 years old at delivery).

For peer review only - http://bmjopen.bmj.com/site/about/guidelines.xhtml

**Explanatory**	% of live births where maternal LSOA history within 10% most deprived LSOAs in England	2006/07 to 2013/14	% of singleton live births recorded in hospital where mother lived in an LSOA that was one of the 10% most deprived LSOAs in England (according to the 2010 Index of Multiple Deprivation - IMD) within the three years prior to delivery.	HES APC (linked by LSOA to 2010 IMD measures)	The LSOA used to derive maternal deprivation could be up to three years out of date at time of delivery. In addition, where women with multiple LSOAs recorded in the look-back period, each LSOA was linked to the 2010 IMD deciles and the minimum decile of deprivation (i.e. most deprived) from all LSOAs recorded was selected.
**Explanatory**	% of live births where child has a complex chronic condition	2006/07 to 2013/14	% of singleton live births with mother-baby linkage where the child had a congenital anomaly - identified where a congenital anomaly- related ICD-10 code[b] was recorded in the child's HES APC record within the first 2 years of life or recorded on a death certificate before the age of 5 years old (to capture children whose congenital anomaly diagnosis was not captured at birth or who were diagnosed later in life).	HES APC	Information on children with congenital anomalies was only available for births with mother-baby record linkage. Therefore this measure was calculated using only singleton live births with linkage available.<br><br>A further nine LAs were excluded as they were missing mother-baby record linkage for more than 35% of singleton live births in at least one financial year between 2006/07 and 2013/14.
**Explanatory**	% of live births with low birth weight	2010/11	% of singleton live births where child had a low birth weight - identified where recorded birthweight < 2500g or a low birth weight- related ICD-10 code (P05.0, P07.0 or P07.1) was recorded in child's HES APC record within seven days of delivery.	HES APC	There considerable variation in quality of birth weight recording by hospitals. Where birth weight was missing in the delivery record but mother-baby linkage was available, we looked for recorded birth weight in the child's birth record and for ICD-10 codes related to low birth weight. The quality of birth weight recording also varied from year to year and therefore we decided to use data only from the 2010/11 year (the mid-point of our study period).<br><br>A further nine LAs were excluded as they were missing a recorded birthweight in the maternal or child (where available) HES APC record at birth for more than 35% of

For peer review only - http://bmjopen.bmj.com/site/about/guidelines.xhtml

					singleton live births between April 2010 and March 2011.
**Explanatory**	% of dependent child households with lone parent	2011	% of households with dependent children (i.e. children aged 0-15 years old), where there is a single parent.	Census 2011 (Table LC1109EW)	
**Explanatory**	Rate of violent crime (per 100 LA residents)	2010/11	The number of violence against the person offences, based on police recorded crime data, per 100 people resident in the LA.	Public Health England Fingertips (Indicator 11202)	This does not capture violent crimes not reported to, or recorded by, the police. In addition, rate of violent crime in city centres with few residents (such as City of London) may be inflated as there will be large numbers of people commuting into these areas who are not counted in the population denominator.

ARA = adversity-related hospital admission; **CLA** = Children Looked After; **HES APC** = Hospital Episode Statistics Admitted Patient Care; **ICD-10** = International Statistical Classification of Diseases and Related Health Problems 10th Revision; **IMD** = Index of Multiple Deprivation; **LA** = local authority; **LSOA** = lower-layer super output area.

[a] We defined history of ARA as any episode of admitted patient care related to substance misuse, mental health problems (including self-harm) or exposure to violence in the look-back period, determined by several non-mutually exclusive lists of ICD-10 codes.[27–30]
[b] Diagnoses of congenital anomalies were identified using a subset of Feudtner et al's ICD-10 code list (i.e. all Q codes).[31]

For peer review only - http://bmjopen.bmj.com/site/about/guidelines.xhtml

Longitudinal modelling

We analysed the relationship over time between the LA-specific, yearly rate of infant entry into care (which were approximately normally distributed) and LA-specific, yearly percentage of singleton live-births with a maternal history of ARA, using linear mixed-effect models with restricted maximum likelihood estimation (for histograms of the outcome by financial year, see Appendix page 7). LA-specific percentage of live births with maternal history of ARA was modelled as a time-varying covariate between 2006/07 and 2013/14 to allow us to examine whether the association under study changed over time. However, the estimated model coefficient for this time-varying covariate could be interpreted as either: (1) the effect of a unit increase in this covariate within the same LA or (2) the effect of a unit increase in this covariate across different LAs. Therefore, we sought to disaggregate these two effects by replacing the original LA-specific variable with two variables: (1) the mean percentage of live births with maternal history of ARA over the study period for each LA (i.e. the between-LA effect), and (2) the difference, within local authorities, between the original LA-specific yearly values and the LA-specific mean (i.e. the within-LA effect). The coefficient for the first variable captures the between-LA effect and the coefficient for the second the within-LA effect.[34] We used Wald χ^2 tests to test the null hypothesis that these two effects were equal. All other LA-level risk factors for entry into care were included in models as non-time varying variables using data from 2010/11 only (i.e. midpoint of study period) as we observed minor variations and this improved model parsimony.

We fitted five models: (1) a ('null') model with only financial year as the explanatory variable; (2) a model with financial year and both LA-specific mean and mean-centred maternal ARA prevalence among live births; (3) a model which included all explanatory measures (as listed in Table 1), including mean and mean-centred maternal ARA prevalence; (4) a model which included all explanatory measures and the original maternal ARA prevalence; and (5) model 4 with an interaction between financial year and maternal ARA prevalence. Models (1) to (4) included random-intercepts for LA and random-slopes for financial year. Model 5 included only random-intercepts for LA as more complex random effects structures did not converge.

The assumption of normality for the level-1 residuals of each model was checked using quantile-quantile plots and histograms and we inspected fixed-effect parameter estimates and standard errors for inflated values that would be symptomatic of multi-collinearity. We used the Akaike Information Criteria (AIC) to assess relative goodness-of-fit and all five models were checked for implausible predicted values. We also performed model-based parametric bootstrap (with 10,000 simulations) to estimate the proportion of variation in the outcome explained by the whole model (i.e. by both fixed- and random-effects) and by only the fixed-effects using formulas for a conditional and marginal pseudo R^2 value, respectively.[35–37] We reported the median marginal and conditional pseudo R^2 values from the bootstrapped samples, along with 95% confidence intervals (using the percentile method). All data management and analysis were carried out using R version 3.5.1.

Patient and Public Involvement

No patients or members of the public were involved in the development or analyses of the current study. However, this study was carried out to inform a wider piece of work: linkage of administrative family court and healthcare data to better understand the healthcare need and health service use of women involved in care proceedings in England, particularly those with a mental health illness. For this larger project, we attended two regular group meetings of mental health service users to discuss the project and the data linkage between administrative family court data and mental health service

For peer review only - http://bmjopen.bmj.com/site/about/guidelines.xhtml

records in England. We also held a focus group with women who have been involved in care proceedings to discuss the study in more detail and gain feedback on the project objectives. These public involvement sessions have helped us to strengthen our research plan, improving the relevance of future findings to the population under study.

RESULTS

For peer review only

For peer review only - http://bmjopen.bmj.com/site/about/guidelines.xhtml

Table 2: Local authority characteristics

LA characteristics (median [min, max])	2006/07 (N = 131)	2007/08 (N = 131)	2008/09 (N = 131)	2009/10 (N = 131)	2010/11 (N = 131)	2011/12 (N = 131)	2012/13 (N = 131)	2013/14 (N = 131)
Number of singleton live births recorded in HES APC	3288 [323, 16,076]	3416 [299, 16,687]	3454 [295, 16,847]	3516 [309, 16,722]	3552 [264, 16,734]	3550 [298, 17,132]	3440 [333, 16,932]	3415 [303, 16,438]
Rate of infant entry to care (per 10,000)	72.76 [0.00, 240.00]	66.25 [8.83, 184.70]	72.99 [4.62, 197.18]	79.19 [0.00, 280.26]	81.89 [4.51, 195.74]	90.16 [10.26, 253.56]	93.05 [0.00, 318.51]	90.14 [13.94, 269.50]
LA Population size	56,455 [8000, 313,364]	56,783 [8140, 315,580]	57,453 [8114, 317,556]	58,339 [7995, 318,654]	58,570 [8079, 320,618]	59,039 [8031, 322,743]	60,307 [7809, 324,341]	60,426 [7795, 326,092]
% of live births with maternal history of ARA	2.73 [0.52, 10.07]	2.89 [0.95, 8.91]	3.15 [1.19, 9.09]	3.66 [0.90, 9.36]	4.33 [1.49, 11.14]	5.21 [1.43, 12.53]	6.15 [1.71, 15.94]	7.01 [3.12, 16.19]
% of live births where mother < 20 years old	7.01 [1.14, 14.50]	6.82 [1.46, 14.13]	6.58 [1.35, 12.82]	6.44 [1.50, 13.89]	5.72 [1.17, 11.89]	5.39 [1.13, 10.77]	5.16 [1.30, 9.93]	4.33 [0.87, 8.49]
% of live births where maternal LSOA history within 10% most deprived LSOAs in England	14.46 [0.00, 60.18]	14.98 [0.00, 60.46]	14.64 [0.00, 61.92]	15.33 [0.00, 60.90]	14.87 [0.00, 60.36]	14.75 [0.11, 61.21]	14.47 [0.00, 61.52]	14.72 [0.24, 60.68]
% of live births where child has a congenital anomaly	1.64 [0.60, 3.34]	1.64 [0.84, 3.25]	1.62 [0.60, 2.74]	1.78 [0.84, 3.68]	1.78 [0.93, 3.19]	1.81 [1.06, 3.38]	1.83 [1.11, 3.61]	1.93 [1.03, 3.22]
% of live births with low birth weight					6.26 [4.22, 9.94]			
% of dependent child households with lone parent					18.31 [9.78, 30.94]			
Rate of violent crime (per 100 LA residents)					1.14 [0.52, 3.17]			

ARA = adversity-related hospital admission; **HES APC** = Hospital Episode Statistics Admitted Patient Care; **LA** = local authority; **LSOA** = lower-layer super output area.
Note: the median LA value is presented as many of the explanatory measures are non-normally distributed.

For peer review only - http://bmjopen.bmj.com/site/about/guidelines.xhtml

Table 2 summarises the LA characteristics for the study cohort by financial year. The median number of residents per LA increased over time (56455 residents in 2006/07 vs 60426 in 2013/14), while the median number of live births (3288 in 2006/07 vs 3415 in 2013/14) and the median percentage of live births where maternal LSOA history was within the 10% most deprived English LSOAs (14.46% in 2006/07 vs 14.72% in 2013/14) remained stable. Across all 131 LAs, the median rate of infant entry into care (72.76 per 10,000 in 2006/07 vs 90.14 in 2013/14) and the median percentage of live births with maternal history of ARA (2.73% in 2006/07 vs 7.01 in 2013/14) increased over time. LA-specific rates for both these measures varied substantially each year between LAs. The median percentage of live births where the child had a congenital anomaly also increased over time (1.64% in 2006/07 vs 1.93% in 2013/14), though LA variation decreased over time (min-max: 0.60-3.34% in 2006/07 vs 1.03-3.22% in 2013/14). Both the median percentage of live births to mothers under 20 years old (7.01% in 2006/07 vs 4.33% in 2013/14) and the LA variation in this measure (min-max: 1.14-14.50% in 2006/07 vs 0.87-8.49% in 2013/14) decreased over time. There was variation between LAs in the proportion of live births with low birth weight (min-max: 4.22-9.94%), the rate of violent crime (0.52-3.17 per 100 residents) and the proportion of dependent child households with a lone parent (9.78-30.94%).

[Insert Figure 2 – Figure 2: Modelling the association between LA-specific percentage of live births with maternal history of ARA and LA-specific rate of infant entry into care for 131 English local authorities, over time (2006/07 to 2013/14)]

Figure 2 footnotes:

* Models were adjusted for all other explanatory measures (Table 1).

Notes: the term 'fixed-effects' includes any explanatory measures in the model such as time and maternal history of ARA but does not include random-effects such as random intercepts and random slopes; 95% confidence intervals given in brackets.

[a] Effect where the percentage of live births with maternal history of ARA equal to zero.

[b] Effect in 2006/07.

Modelling the association between rate of infant entry into care and prevalence of maternal adversity-related hospital admissions before birth

Figure 2A displays point estimates and 95% confidence intervals from Models (1) to (5) for the coefficients of the time-varying covariates. Prior to adjustment (i.e. Model 2), a percentage point increase in percentage of live births with maternal history of ARA within the same LA was associated with an extra 2.44 infants, per 10,000, entering care (95% CI: 1.10 to 3.78), while a percentage point increase between two different LAs was associated with an extra 11.63 infants, per 10,000, entering care (8.94 to 14.31). Using Models (1) and (2), we estimated that the percentage of live births with a maternal history of ARA explained 24% (95% CI: 14-35%) of the variation in the rate of infant entry into care between 2006/07 and 2013/14 (Figure 2B). After adjustment for all other explanatory measures (i.e. Model 3), there was insufficient evidence that the effect on the outcome of increases to the percentage of live births with maternal history of ARA within the same LA and the effect on the outcome of increases between different LAs were different (p-value = 0.36). After refitting the adjusted model without disaggregation of the within-LA and between-LA effects (i.e. Model 4), there was evidence that a 1% point increase in the percentage of live births with maternal history of ARA, either within the same LA or between two different LAs, was associated with an extra 2.56

For peer review only - http://bmjopen.bmj.com/site/about/guidelines.xhtml

infants per 10,000 entering care (1.31 to 3.82) over a 12-month period, holding all other model covariates constant. Finally, we explored whether the effect of an increase within the same LA or between two different LAs in percentage of live births with maternal history of ARA varied over the study period (i.e. Model 5). There was evidence that the magnitude of the association between the percentage of live births with maternal history of ARA (by LA, over the study period) and rate of infant entry into care increased over time between 2006/07 and 2013/14 (interaction coefficient estimate: 0.44, 95% CI: 0.15 to 1.44), as seen in Figure 3. (For full model results, see Appendix pages 8-10).

[Insert Figure 3 here - Figure 3: Exploring changes in the association between the LA-specific percentage of live births with maternal history of ARA and LA-specific rate of infant entry into care between 2006/07 and 2013/14]

DISCUSSION

We found that increases either between LAs or within the same LA over time in the rate of infant entry into care were associated with an increased prevalence of maternal history of adversity related hospital admission (ARA). Evidence for this association persisted even after controlling for other, potentially confounding, LA-level risk factors for entry to care. The magnitude of the increase in rate of infant entry into care per percentage point increase in the percentage of live births with maternal history of ARA increased over time, particularly from 2009/10 onwards (Figure 3). We estimated that the percentage of live births with maternal history of ARA alone explained between 14-35% of the LA variation in rate of infant entry into care over the study period. The final model, with all covariates included, explained 47-60% of this variation.

Strengths

This is the first study to account for maternal health-related risk factors when examining variation among English LA rates of infant entries into care. A key strength of this study is the breadth of information on mothers and children included in the models. Six out of nine of our measures were derived using two national, longitudinal databases (HES APC and CLA), each with person-level records enabling follow up through health and social care services throughout England over time. In particular, HES APC captures diagnoses via ICD-10 codes, allowing us to identify adversity-related healthcare need among mothers up to three years before delivery (i.e. maternal history of ARA) that was sufficiently severe to be recorded during a hospital admission. Many of the ARA codes have been previously validated in other populations.[38–41] Another strength is the inclusion of multiple risk factors for maltreatment and infant entry into care in statistical modelling. We designed our modelling approach to balance model parsimony with adjustment for confounders that were supported by external evidence, which were relevant to policy and measurable. We further preserved model parsimony by allowing only our main exposure (maternal history of ARA) to vary over time, while fixing all other model covariates at their 2010/11 values. Most of the explanatory measures in this final model were derived from administrative hospital records (HES APC), highlighting the importance of considering indicators for parental health near to birth when exploring LA variation in rate of infant entry to care.

Limitations

For peer review only - http://bmjopen.bmj.com/site/about/guidelines.xhtml

The main limitation of this study sits with its ecological design. While we have found that increases in the percentage of live births with maternal history of ARA are associated with increases in the rate of infant entry to care over a given area, we cannot examine, at an individual level, whether children born to women with a history of adversity-related admission prior to birth were more likely to be placed into care during infancy. However, there is currently no English database containing mother-to-baby linked healthcare data with onward linkage to information on children's social care outcomes. We, therefore, studied the association between maternal adversity prior to birth and infant entry to care at an LA level. Another limitation is that we did not explore the effect of increases to LA prevalence of maternal history of hospital admissions related to particular types (or combination of types) of adversity prior to birth. We took this decision partly to avoid increasing the risk of Type I error inflation due to excessive statistical testing (relative to our sample size) and partly due to a number of LAs having non-disclosable values (< 10) for this measure when stratified by type (or combination of types) of adversity. There is also a lack of information on 'supply-side' factors to infant entry to care, such as funding for early intervention programmes and availability of foster care placements, of sufficient quality for research at the LA level for the whole of England. A further limitation is that we cannot distinguish whether increases over time in the percentage of live births with maternal history of ARA reflect a true increase or are partly explained by nationwide changes in coding practices, although adversity admissions appear to be increasing particularly among younger women.[42] Finally, we were limited to maternal and not paternal adversity-related hospital admission as it is not possible to identify fathers in HES APC. We were also restricted to hospital indicators of adversity as primary care indicators cannot be linked to local authority.

Implications of the study findings

Until now, there has been a paucity of evidence on the association between local variation in demand for child protection intervention in England and parental health indicators that are associated with diminished parenting. Most prior studies have instead focussed on the quantifying the relationship between variation in demand and poverty.[16,43,44] We found that hospital admissions related to adversity such as substance misuse, exposure to violence, and mental health problems explained a substantial proportion of LA variation in the rate of infant entry into care, even after adjustment for maternal deprivation. The prevalence and contribution of this risk factor also appears to be increasing with time. These admissions present opportunities to respond to adversity-related healthcare need prior to pregnancy and birth to improve maternal health and potentially mitigate infant entry into care. However, the success of early intervention in a healthcare setting will rely upon effective multi-agency collaboration between health, social care and third sector organisations within LAs, which was a key recommendation from the Care Crisis Review into the challenges facing the children's social care sector in England.[2] Services should also provide a relationship-based and flexible approach to support service users who initially fail to engage and to mitigate issues arising from distrust of professionals.[45,46] Further, parents and parents-to-be who are subject to children's social care involvement often have a relatively short window in which to make changes; therefore, services targeted to this population must offer timely access to ensure support is available within the timeframes of children's social care assessments and family court proceedings.

We saw increased acceleration over time in the average LA percentage of live births with maternal history of adversity-related hospital admission and increased magnitude over time in the effect of increases to LA percentage of live births with history of maternal adversity-related hospital admission on the rate of infant entry into care, which coincided with the introduction of cuts to

For peer review only - http://bmjopen.bmj.com/site/about/guidelines.xhtml

central government funding for LAs.[47] These cuts led many LAs to decrease spend on early intervention, youth services, and some public health programmes.[48,49] However, it was beyond the scope of this work to investigate the effect of austerity on changes to the association between the percentage of live births with maternal history of ARA and rate of infant entry into care over time.

This study highlights the importance of establishing a linked parent-child healthcare data resource with onward linkage to CLA and other social care data, to enable more robust evaluation of the association between maternal ARA and other health indicators and infant entry into care to inform preventive interventions. Such data linkages would be vital to inform policy strategies aimed at improving women's health, wellbeing, and reproductive rights, and potentially reduce infant entries to care.

For peer review only

For peer review only - http://bmjopen.bmj.com/site/about/guidelines.xhtml

REFERENCES

1 Department for Education. Statistics: looked-after children. 2019.https://www.gov.uk/government/collections/statistics-looked-after-children (accessed 26 Sep 2019).

2 Family Rights Group. Care Crisis Review: options for change. 2018.https://www.frg.org.uk/images/Care_Crisis/CCR-FINAL.pdf (accessed 1 Oct 2019).

3 Oranga Tamariki. Babies and children entering Oranga Tamariki care. 2019.https://www.orangatamariki.govt.nz/assets/Uploads/OIA-responses/children-in-care/20190628-Babies-and-children-entering-Oranga-Tamariki-care.pdf (accessed 17 Oct 2019).

4 Woods R, Henderson G. Changes in out of home care and permanence planning among young children in Scotland, 2003 to 2017. *Adopt Foster* Published Online First: 2018. doi:10.1177/0308575918790435

5 Marsh CA, Browne J, Taylor J, *et al.* Characteristics and outcomes of newborns entered who entered into care (EIC) within 7 days of birth in NSW, Australia. *Child Youth Serv Rev* Published Online First: 2017. doi:10.1016/j.childyouth.2017.08.005

6 Australian Institute of Health and Welfare. Data tables: Child protection Australia 2017-18. 2019.https://www.aihw.gov.au/reports/child-protection/child-protection-australia-2017-18/data (accessed 17 Oct 2019).

7 Children's Bureau. Adoption & Foster Care Statistics. https://www.acf.hhs.gov/cb/research-data-technology/statistics-research/afcars (accessed 22 Oct 2019).

8 Ghertner R, Waters A, Radel L, *et al.* The role of substance use in child welfare caseloads. *Child Youth Serv Rev* 2018;**90**:83–93. doi:10.1016/j.childyouth.2018.05.015

9 Russell J, Macgill S. Demographics, policy, and foster care rates; A Predictive Analytics Approach. *Child Youth Serv Rev* 2015;**58**:118–26. doi:10.1016/j.childyouth.2015.09.009

10 Naughton AM, Maguire SA, Mann MK, *et al.* Emotional, behavioral, and developmental features indicative of neglect or emotional abuse in preschool children a systematic review. JAMA Pediatr. 2013. doi:10.1001/jamapediatrics.2013.192

11 Hughes K, Bellis MA, Hardcastle KA, *et al.* The effect of multiple adverse childhood experiences on health: a systematic review and meta-analysis. *Lancet Public Heal* Published Online First: 2017. doi:10.1016/S2468-2667(17)30118-4

12 Children Act 1989, s.17. England & Wales: 1989. http://www.legislation.gov.uk/ukpga/1989/41/section/17

13 Stith SM, Liu T, Davies LC, *et al.* Risk factors in child maltreatment: A meta-analytic review of the literature. Aggress. Violent Behav. 2009. doi:10.1016/j.avb.2006.03.006

14 Mulder TM, Kuiper KC, van der Put CE, *et al.* Risk factors for child neglect: A meta-analytic review. *Child Abuse Negl* 2018;**77**:198–210. doi:https://doi.org/10.1016/j.chiabu.2018.01.006doi

15 Simkiss DE, Stallard N, Thorogood M. A systematic literature review of the risk factors associated with children entering public care. *Child Care Health Dev* 2013;**39**:628–42. doi:10.1111/cch.12010

16 National Audit Office. Pressures on children's social care.

For peer review only - http://bmjopen.bmj.com/site/about/guidelines.xhtml

2019.https://www.nao.org.uk/report/pressures-on-childrens-social-care/ (accessed 10 Apr 2019).

17 Guttmann A, Blackburn R, Amartey A, *et al.* Long-term mortality in mothers of infants with neonatal abstinence syndrome: A population-based parallel-cohort study in England and Ontario, Canada. *PLOS Med* Published Online First: 2019. doi:https://doi.org/10.1371/journal.pmed.1002974

18 O'Donnell M, Maclean M, Sims S, *et al.* Entering out-of-home care during childhood: Cumulative incidence study in Canada and Australia. *Child Abus Negl* Published Online First: 2016. doi:10.1016/j.chiabu.2016.07.011

19 Jenkins BQ, Tilbury C, Hayes H, *et al.* Factors associated with child protection recurrence in Australia. *Child Abus Negl* Published Online First: 2018. doi:10.1016/j.chiabu.2018.05.002

20 White OG, Hindley N, Jones DPH. Risk factors for child maltreatment recurrence: An updated systematic review. Med. Sci. Law. 2015. doi:10.1177/0025802414543855

21 Office for National Statistics. Estimates of the population for the UK, England and Wales, Scotland and Northern Ireland: mid-2001 to mid-2017 detailed time series. 2018.https://www.ons.gov.uk/peoplepopulationandcommunity/populationandmigration/populationestimates/datasets/populationestimatesforukenglandandwalesscotlandandnorthernireland (accessed 21 Sep 2018).

22 Mc Grath-Lone L, Harron K, Dearden L, *et al.* Data Resource Profile: Children Looked After Return (CLA). *Int J Epidemiol* 2016;**45**:716-717f. doi:10.1093/ije/dyw117

23 Herbert A, Wijlaars L, Zylbersztejn A, *et al.* Data Resource Profile: Hospital Episode Statistics Admitted Patient Care (HES APC). *Int J Epidemiol* 2017;**46**:1093-1093i. doi:10.1093/ije/dyx015

24 World Health Organization. International Statistical Classification of Diseases and Related Health Problems - 10th Revision. Geneva: 2011.

25 Ministry of Housing C& LG. English indices of deprivation 2010: all domains, all sub domains and supplementary indices. 2011.https://www.gov.uk/government/statistics/english-indices-of-deprivation-2010 (accessed 29 Apr 2019).

26 Harron K, Gilbert R, Cromwell D, *et al.* Linking Data for Mothers and Babies in De-Identified Electronic Health Data. *PLoS One* 2016;**11**:e0164667. doi:10.1371/journal.pone.0164667

27 Nilsson SF, Laursen TM, Hjorthøj C, *et al.* Risk of psychiatric disorders in offspring of parents with a history of homelessness during childhood and adolescence in Denmark: a nationwide, register-based, cohort study. *Lancet Public Heal* 2017;**2**:E541–50. doi:10.1016/S2468-2667(17)30210-4

28 Herbert A, Gilbert R, González-Izquierdo A, *et al.* Violence, self-harm and drug or alcohol misuse in adolescents admitted to hospitals in england for injury: A retrospective cohort study. *BMJ Open* 2015;**5**. doi:10.1136/bmjopen-2014-006079

29 Fone D, Morgan J, Fry R, *et al.* Change in alcohol outlet density and alcohol-related harm to population health (CHALICE): a comprehensive record-linked database study in Wales. *Public Heal Res* Published Online First: 2016. doi:10.3310/phr04030

30 González-Izquierdo A, Woodman J, Copley L, *et al.* Variation in recording of child maltreatment in administrative records of hospital admissions for injury in England, 1997-2009. *Arch Dis Child* Published Online First: 2010. doi:10.1136/adc.2009.180216

31 Feudtner C, Feinstein JA, Zhong W, *et al.* Pediatric complex chronic conditions classification

For peer review only - http://bmjopen.bmj.com/site/about/guidelines.xhtml

system version 2: Updated for ICD-10 and complex medical technology dependence and transplantation. *BMC Pediatr* 2014;**14**. doi:10.1186/1471-2431-14-199

32 Public Health England. Public Health England Fingertips Tool. https://fingertips.phe.org.uk/ (accessed 25 Jul 2019).

33 Office for National Statistics. 2011 Census: Local Characteristics data tables. 2011.https://www.nomisweb.co.uk/census/2011/local_characteristics (accessed 12 Mar 2019).

34 Rabe-Hesketh S, Skrondal A. *Multilevel and Longitudinal Modeling Using Stata*. 3rd ed. College Station, Texas: : Stata Press 2012.

35 Nakagawa S, Schielzeth H. A general and simple method for obtaining R2 from generalized linear mixed-effects models. *Methods Ecol Evol* Published Online First: 2013. doi:10.1111/j.2041-210x.2012.00261.x

36 Johnson PCD. Extension of Nakagawa & Schielzeth's R2GLMM to random slopes models. *Methods Ecol Evol* Published Online First: 2014. doi:10.1111/2041-210X.12225

37 Bartoń K. Package 'MuMIn': Multi-Model Inference. *Cran-R* Published Online First: 2018.www.cran.r-project.org/web/packages

38 Kim H, Smith EG, Stano CM, *et al.* Validation of key behaviourally based mental health diagnoses in administrative data: Suicide attempt, alcohol abuse, illicit drug abuse and tobacco use. *BMC Health Serv Res* Published Online First: 2012. doi:10.1186/1472-6963-12-18

39 Davis KAS, Sudlow CLM, Hotopf M. Can mental health diagnoses in administrative data be used for research? A systematic review of the accuracy of routinely collected diagnoses. *BMC Psychiatry* Published Online First: 2016. doi:10.1186/s12888-016-0963-x

40 Swain RS, Taylor LG, Braver ER, *et al.* A systematic review of validated suicide outcome classification in observational studies. *Int J Epidemiol* Published Online First: 2019. doi:10.1093/ije/dyz038

41 Gonzalez-Izquierdo A, Ward A, O'Donnell M, *et al.* Cross-country comparison of victimisation-related injury admission in children and adolescents in England and Western Australia. *BMC Health Serv Res* Published Online First: 2013. doi:10.1186/1472-6963-13-260

42 Blackburn RM, Herbert A, Wijlaars L, *et al.* Trends in Hospital Admissions for Nonfatal Adversity-Related Injury among Youths in England, 2002-2016. JAMA Pediatr. 2018. doi:10.1001/jamapediatrics.2018.2516

43 Emmott EH, Mc Grath-Lone L, Harron K, *et al.* Explaining local variation in referrals from health services to children's social care in England 2013–16: a study using 'children in need' administrative data. *J Public Health (Bangkok)* Published Online First: 2019. doi:10.1093/pubmed/fdz050

44 Bywaters P, Brady G, Bunting L, *et al.* Inequalities in English child protection practice under austerity: A universal challenge? *Child Fam Soc Work* Published Online First: 2018. doi:10.1111/cfs.12383

45 Canvin K, Jones C, Marttila A, *et al.* Can I risk using public services? Perceived consequences of seeking help and health care among households living in poverty: Qualitative study. *J Epidemiol Community Health* Published Online First: 2007. doi:10.1136/jech.2006.058404

46 Mason C, Robertson L, Broadhurst K. Pre-birth assessment and infant removal at birth: experiences and challenges. 2019.https://www.nuffieldfoundation.org/wp-

For peer review only - http://bmjopen.bmj.com/site/about/guidelines.xhtml

content/uploads/2019/12/Literature-review_Born-into-Care_Dec-2019.pdf

47 National Audit Office. Financial sustainability of local authorities 2018. 2018.https://www.nao.org.uk/wp-content/uploads/2018/03/Financial-sustainabilty-of-local-authorites-2018.pdf (accessed 22 Oct 2019).

48 British Medical Association. Feeling the squeeze. The local impact of cuts to public health budgets in England. 2018.https://www.bma.org.uk/collective-voice/policy-and-research/public-and-population-health/public-health-budgets (accessed 22 Oct 2019).

49 Kelly E, Lee T, Sibieta L, *et al.* Public Spending on Children in England: 2000 to 2020. 2018.https://www.childrenscommissioner.gov.uk/wp-content/uploads/2018/06/Public-Spending-on-Children-in-England-CCO-JUNE-2018.pdf (accessed 1 Oct 2019).

For peer review only

For peer review only - http://bmjopen.bmj.com/site/about/guidelines.xhtml

COMPETING INTERESTS

We declare no competing interests.

FUNDING

Linda Wijlaars, Ruth Gilbert, Rachel J Pearson and Stuart Bedston are supported by funding from the Nuffield Foundation, grant reference number KID/42838. Ruth Gilbert also receives funding from Health Data Research UK. Matthew A Jay is supported by funding from the Medical Research Council through the UCL Birkbeck Doctoral Training Partnership (MR/R502248/1). This work is supported by the NIHR GOSH BRC. This research benefits from and contributes to the NIHR Children and Families Policy Research Unit, but was not commissioned by the National Institute for Health Research (NIHR) Policy Research Programme. The views expressed are those of the author(s) and not necessarily those of the Nuffield Foundation, the NHS, the NIHR or the Department of Health.

Role of the funding source

The funder had no role in the study design, data collection, analysis, interpretation, or writing of the report. The corresponding author had full access to all the data in the study and had final responsibility for the decision to submit for publication.

ETHICAL APPROVAL

Both HES APC and CLA data were pseudonymised before we received them and all other data were already publicly available and anonymised, therefore this study did not require ethical approval.

ACKNOWLEDGMENTS

We would like to thank Dr Ania Zylbersztejn and Dr Katie Harron for their past work on an extract of Hospital Episode Statistics Admitted Patient Care to identify congenital anomaly diagnoses among children and to link mother and child records, respectively. This work uses data provided by individuals and collected by local authorities, the Department for Education and the NHS as part of their care and support.

AUTHOR CONTRIBUTIONS

RJP carried out all data analysis and produced the first draft of this manuscript. RG contributed critically to the formulation of the research question and study design, with input from all other authors (RJP, MAJ, LW, BDS, SS and SB). LW and MAJ provided expertise on the HES APC and CLA data extracts, respectively. MAJ also reviewed content making reference to family law. SS reviewed the literature for validated ICD-10 codes to identify adversity-related healthcare use. BDS provided statistical guidance. All authors (RJP, MAJ, LW, BDS, SS, SB and RG) provided critical revision to the initial manuscript and approved the final version to publish.

DATA SHARING STATEMENT

For peer review only - http://bmjopen.bmj.com/site/about/guidelines.xhtml

For peer review only

No additional data on HES APC and the CLA return are available. All other data used in this study may be accessed freely online:

1) http://www.ons.gov.uk/ons/publications/all-releases.html?definition=tcm%3A77-22371,
2) https://www.nomisweb.co.uk/census/2011/lc1109ew, and
3) https://fingertips.phe.org.uk/search/violence#page/6/gid/1/pat/6/par/E12000008/ati/102/are/E06000039.

For peer review only

For peer review only - http://bmjopen.bmj.com/site/about/guidelines.xhtml

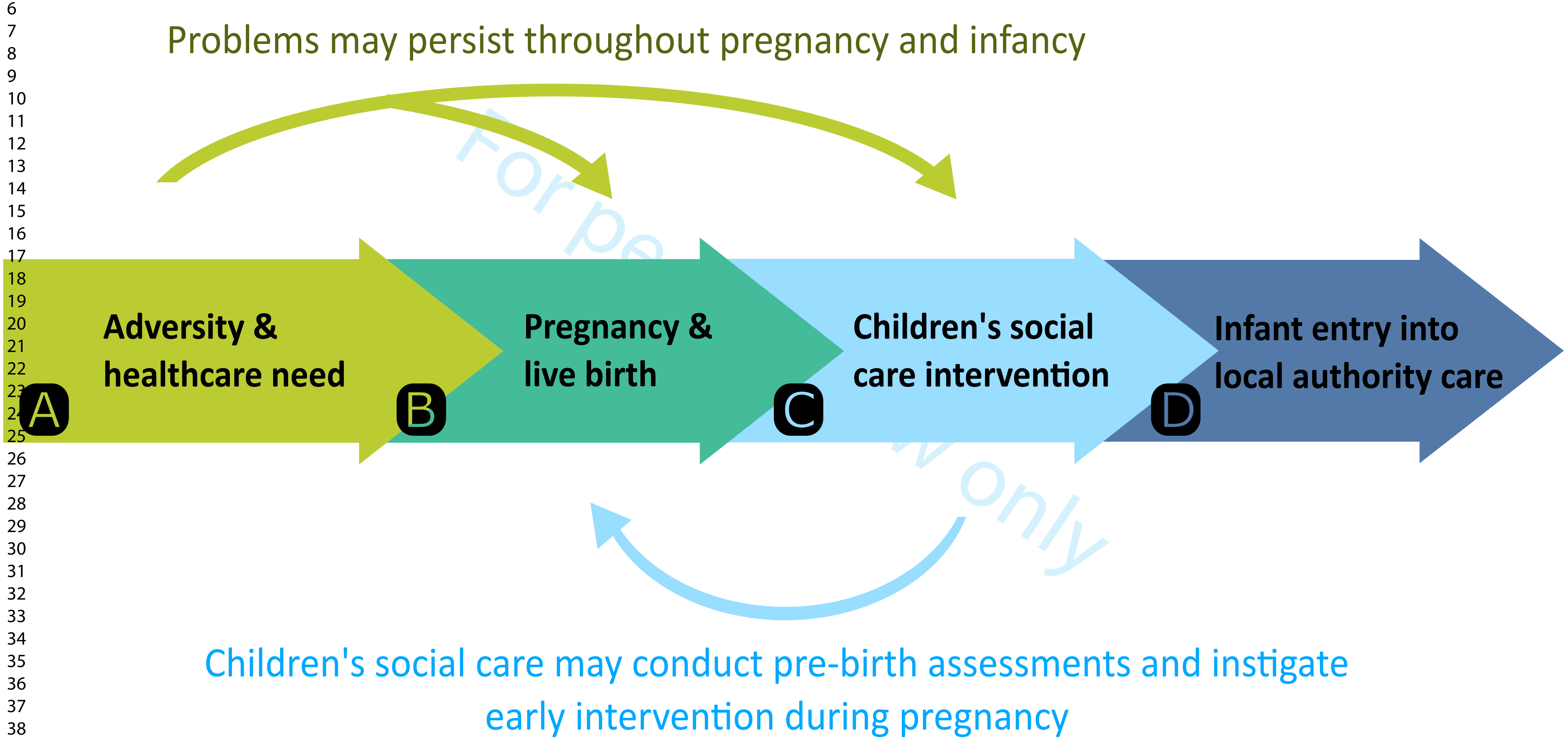

For peer review only - http://bmjopen.bmj.com/site/about/guidelines.xhtml

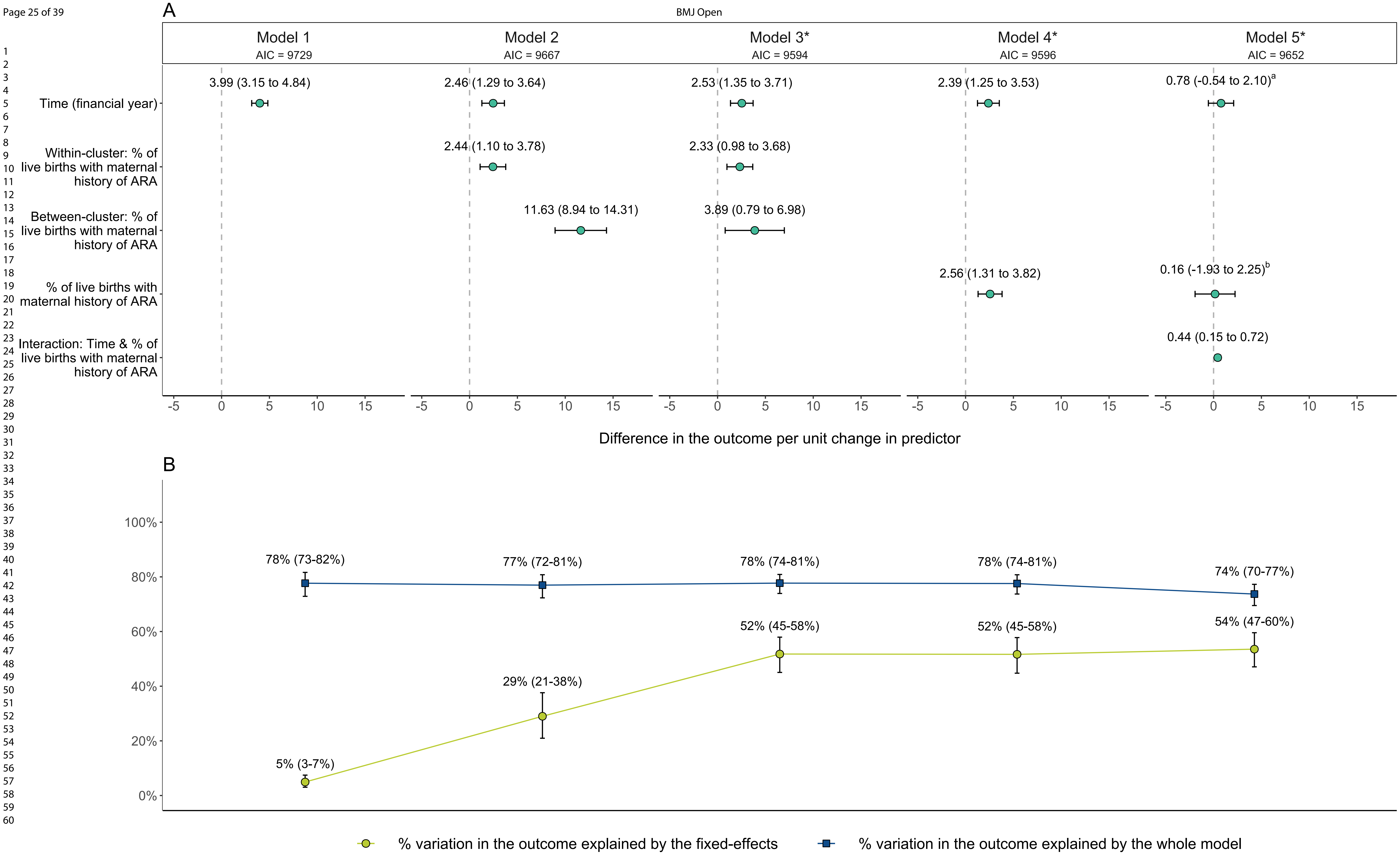
A
Model 1
AIC = 9729
Model 2
AIC = 9667
Model 3*
AIC = 9594
Model 4*
AIC = 9596
Model 5*
AIC = 9652
Time (financial year)
Within-cluster: % of live births with maternal history of ARA
Between-cluster: % of live births with maternal history of ARA
% of live births with maternal history of ARA
Interaction: Time & % of live births with maternal history of ARA
3.99 (3.15 to 4.84)
2.46 (1.29 to 3.64)
2.53 (1.35 to 3.71)
2.39 (1.25 to 3.53)
0.78 (-0.54 to 2.10)a
2.44 (1.10 to 3.78)
2.33 (0.98 to 3.68)
11.63 (8.94 to 14.31)
3.89 (0.79 to 6.98)
2.56 (1.31 to 3.82)
0.16 (-1.93 to 2.25)b
0.44 (0.15 to 0.72)
-5
0
5
10
15
Difference in the outcome per unit change in predictor
B
100%
80%
60%
40%
20%
0%
78% (73-82%)
77% (72-81%)
78% (74-81%)
78% (74-81%)
74% (70-77%)
5% (3-7%)
29% (21-38%)
52% (45-58%)
52% (45-58%)
54% (47-60%)
% variation in the outcome explained by the fixed-effects
% variation in the outcome explained by the whole model

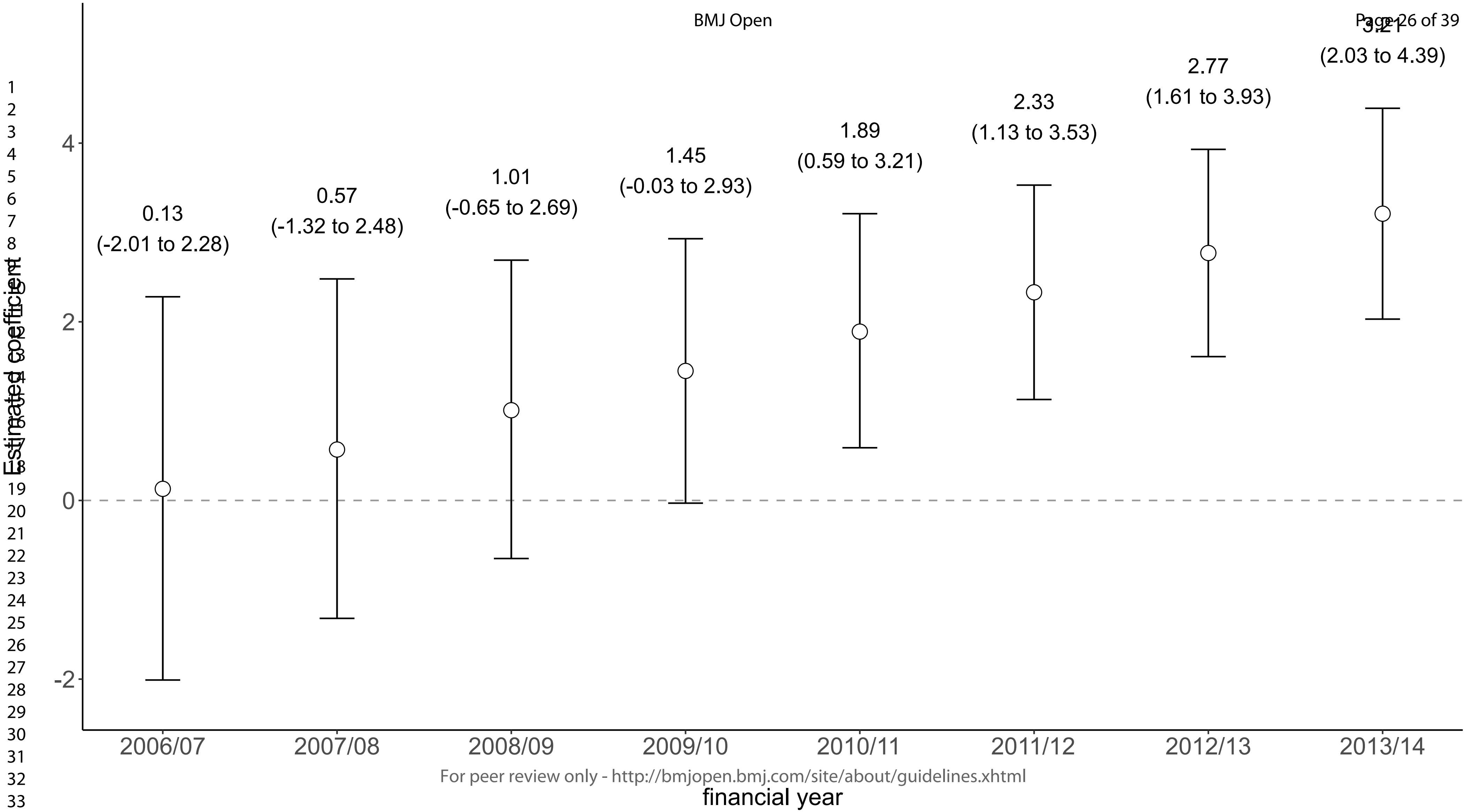
0.13
(-2.01 to 2.28)
0.57
(-1.32 to 2.48)
1.01
(-0.65 to 2.69)
1.45
(-0.03 to 2.93)
1.89
(0.59 to 3.21)
2.33
(1.13 to 3.53)
2.77
(1.61 to 3.93)
3.21
(2.03 to 4.39)
Estimated coefficient
4
2
0
-2
2006/07
2007/08
2008/09
2009/10
2010/11
2011/12
2012/13
2013/14
financial year

Supplementary appendix

Contents

Figure A1: An ecological framework of child maltreatment 2
Figure A2: A flow diagram of the Children Looked After (CLA) cohort selection 3
Missing data: CLA return 3
Figure A3: A flow diagram of the Hospital Episode Statistics Admitted Patient Care (HES APC) cohort selection 4
Missing data: HES APC 4
Table A1: Censored values in measures derived from HES APC 5
Table A2: ICD-10 code lists to identify an adversity-related hospital admission (ARA) 6
Figure A4: The distribution of rate of infant entry to care over the study period 7
Table A3 - Linear mixed-effect modelling results (Models 1-4) 8
Table A4 – Linear mixed-effect modelling results (Model 5) 10
Table A5: Conditional coefficient estimates for percentage of live births with maternal history of adversity-related hospital admissions, by financial year (Model 5) 11

For peer review only

For peer review only - http://bmjopen.bmj.com/site/about/guidelines.xhtml

Figure A1: An ecological framework of child maltreatment

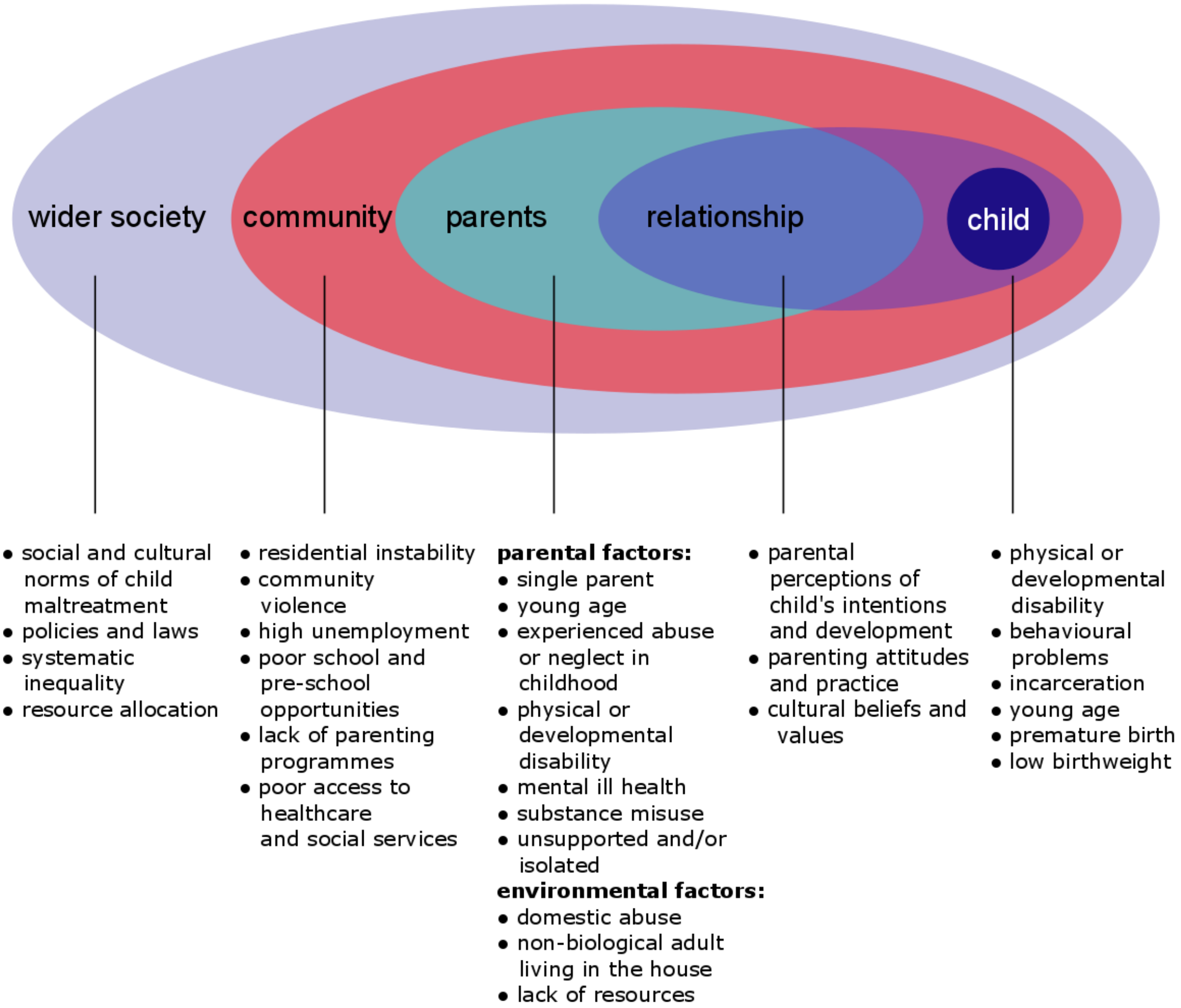

Recognising the ecological and interconnected nature of risk-factors for child maltreatment at different ecological levels, many have applied an ecological framework to this field of study over the years.[1–3] The ecological framework below builds upon previous diagrams and incorporates evidence from several systematic reviews looking at risk-factors for child maltreatment. [4–6]

For peer review only - http://bmjopen.bmj.com/site/about/guidelines.xhtml

Figure A2: A flow diagram of the Children Looked After (CLA) cohort selection

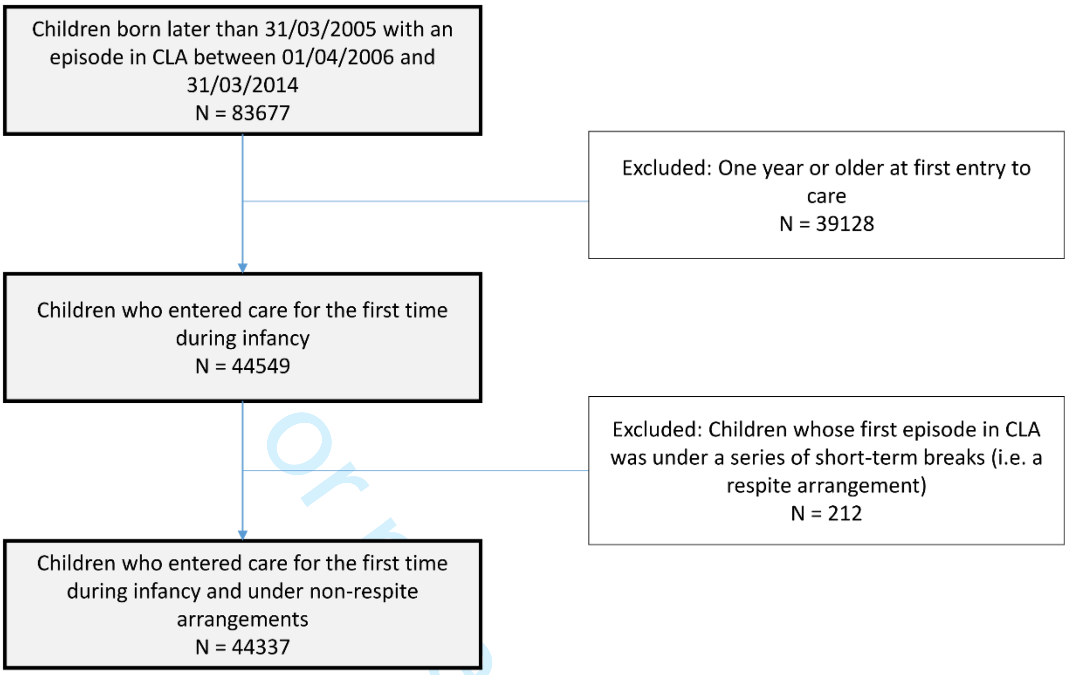

Missing data: CLA return

Adhering to the Department for Education's statistical disclosure rules, we censored any counts less than 10 before outputting them from our secure data environment. Of our 1048 LA counts of infants entering care, 60 (5.7%) had to be censored before we could output them from UCL's secure data environment. We imputed these censored values with a randomly selected number from 0 to 9 before analysis (random seed set to 1234).

For peer review only - http://bmjopen.bmj.com/site/about/guidelines.xhtml

Figure A3: A flow diagram of the Hospital Episode Statistics Admitted Patient Care (HES APC) cohort selection

We derived two cohorts using HES APC (please note that the extract of HES APC used only included individuals with dates of birth available):

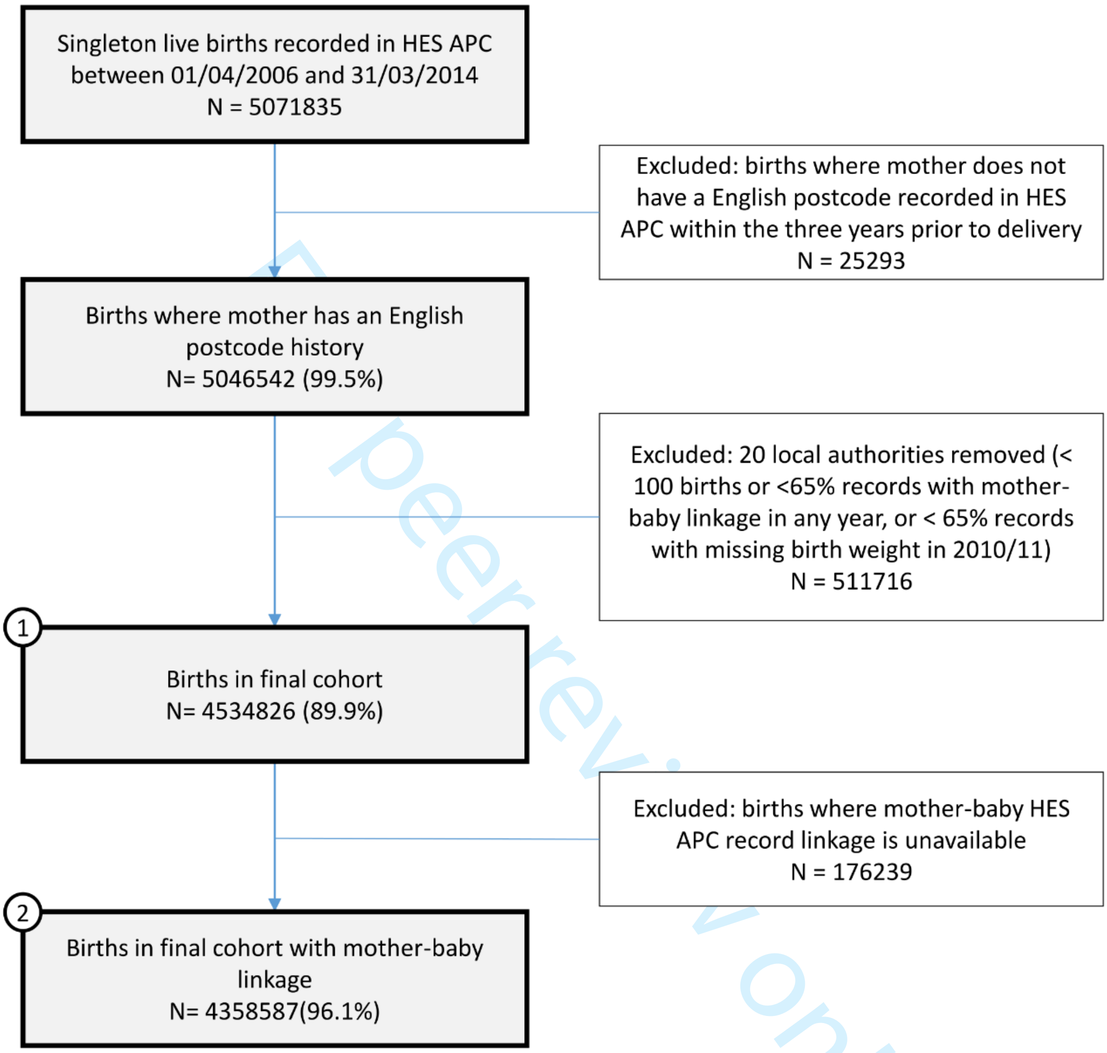

For peer review only

Missing data: HES APC

Adhering to the Department for Health and Social Care's statistical disclosure rules, we censored any values derived from HES APC where the underlying counts were less than five before outputting them from our secure data environment. After outputting, we imputed censored counts with a randomly selected number from 0 to 4 (random seed set to 1234).

For peer review only - http://bmjopen.bmj.com/site/about/guidelines.xhtml

Table A1: Censored values in measures derived from HES APC

Explanatory measure	Number of values	Number of values less than five (%)
% of live births with maternal history of ARA	1048	0
% of live births where mother < 20 years old	1048	0
% of live births where maternal LSOA history within 10% most deprived LSOAs in England	1048	24 (2.29%)
% of live births where child has a complex chronic condition	1048	1 (0.10%)
% of live births with low birth weight	1048	0

For peer review only

Table A2: ICD-10 code lists to identify an adversity-related hospital admission (ARA)

ICD-10 codes	Source
Mental Health disorders:	
Behavioural and emotional disorder<br>F90-F95, F98	Nilsson, 2017
Neurotic, stress-related, and somatoform disorder<br>F40-F45, F48	
Schizophrenia , Personality disorder, or Bipolar disorder<br>F20, F30-F33, F60-F62	
Depressive episode or disorder<br>F32-F33	
Substance use disorder<br>F10-F16, F18-F19	
Other mental health disorder<br>F00-F09, F21-F25, F28, F34, F38-F39, F50-F55, F59, F63-F66, F68-F73, F79-F84, F88-F89, F99	
Intentional self-harm<br>X60-X84	
History of self-harm<br>Z91.5 (we also included Y87.0 – sequelae of intentional self-harm)	Herbert, 2015
Substance misuse:	
Alcohol misuse<br>E51.2, T51.0, X45.0-X45.2, X45.4, X45.6, X45.8, X45.9, X65.0-X65.2, X65.4, X65.5, X65.6, X65.8, X65.9, Y15.0, Y15.2, Y15.4, Y15.8, Y15.9, Y90.0-Y91.3, Y91.9, Z50.2, Z71.4, Z72.1	Fone, 2016
Alcohol and other substance misuse<br>E24.4, F10-F16, F18-F19, G31.2, G62.1, G72.1, I42.6, K29.2, K70.0-K70.9, K85.2, K86.0, O35.4, R78.0-R78.5, T36-T50 (we did not exclude T50.6, however), X40-X44, X46-X49, X69, Y10-Y14, Y16-Y19, Z04.0, Z50.3, Z71.5, Z72.2, T51	Herbert, 2015
Exposure to violence:	
Assault<br>X85-X99, Y01-Y05, Y08-Y09	Herbert, 2015
Maltreatment<br>T73-T74, Y06-Y07	
Injury: undetermined cause<br>Y20-Y34, Z04.5 (we excluded Z04.8)	

For peer review only - http://bmjopen.bmj.com/site/about/guidelines.xhtml

Figure A4: The distribution of rate of infant entry to care over the study period

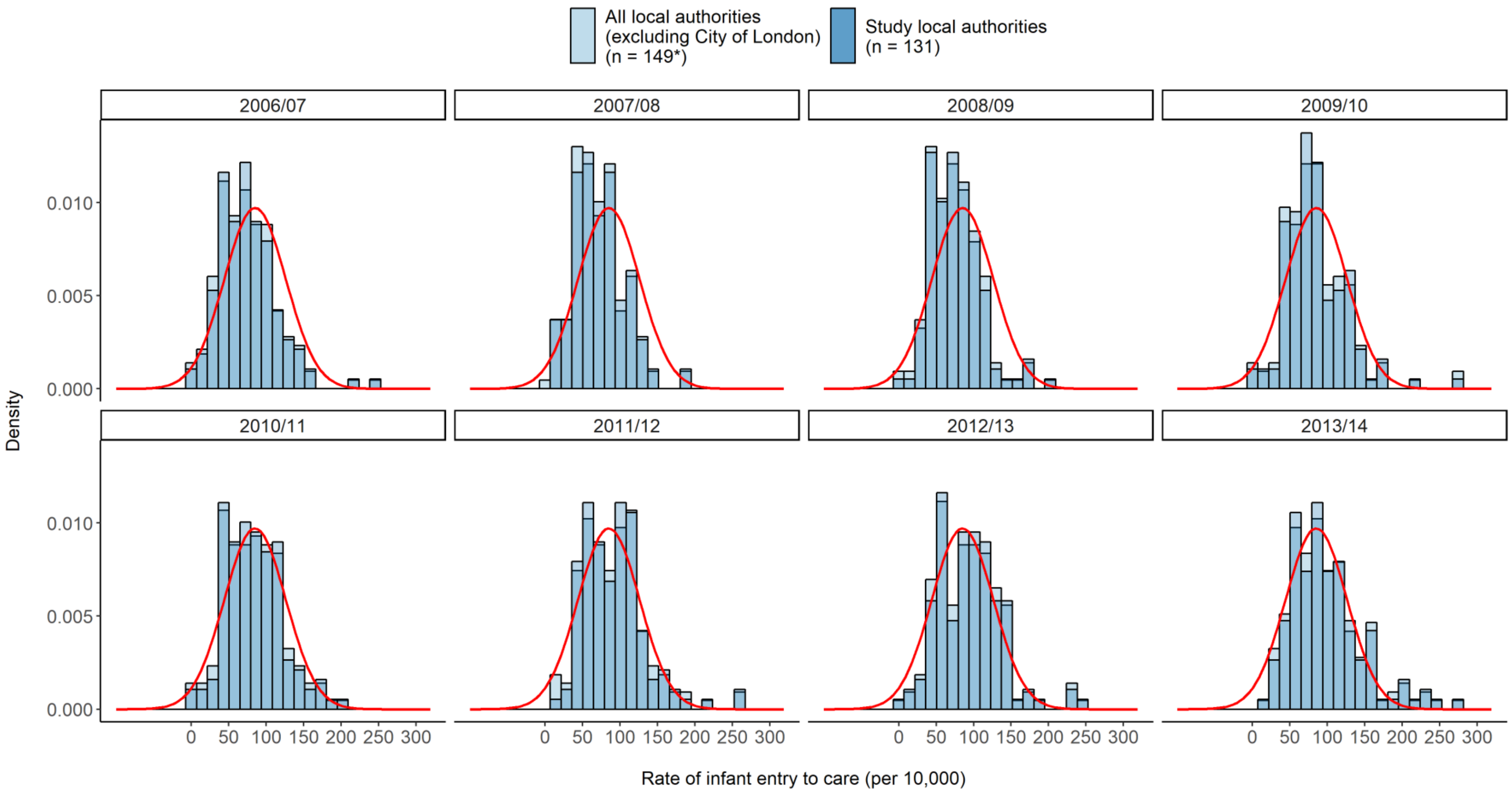

* There were 151 local authorities in 2006/07; however, the Isles of Scilly did not have any children in their care over the study period.
Data source: Children Looked After return, Department for Education.

Table A3 - Linear mixed-effect modelling results (Models 1-4)

Model parameter	Model 1		Model 2		Model 3		Model 4		Type of model parameter
	Estimate	95% C.I.	Estimate	95% C.I.	Estimate	95% C.I.	Estimate	95% C.I.	
Time (e.g. financial year Apr-March)	3.99	3.15 to 4.84	2.46	1.29 to 3.64	2.53	1.35 to 3.71	2.39	1.25 to 3.53	fixed-effect
% of live births with maternal history of ARA			(within-LA) 2.44 (between-LA) 11.63	1.10 to 3.78 8.94 to 14.31	2.33 3.89	0.98 to 3.68 0.79 to 6.98	(overall) 2.56	1.31 to 3.82	fixed-effect
% of live births where mother < 20 years old					6.77	4.50 to 9.03	7.25	5.22 to 9.28	fixed-effect
% of live births where maternal postcode history within 10% most deprived LSOAs in England					0.01	-0.35 to 0.37	0.04	-0.31 to 0.39	fixed-effect
% of live births where child has a complex chronic condition					13.42	4.55 to 22.30	14.22	5.51 to 22.93	fixed-effect
% of live births with low birth weight					1.28	-2.93 to 5.48	1.00	-3.16 to 5.17	fixed-effect
LA population size (000s)					-0.08	-0.14 to -0.02	-0.08	-0.14 to -0.02	fixed-effect
% of dependent child households with lone parent					1.87	0.65 to 3.09	1.96	0.76 to 3.17	fixed-effect
Rate of violent crime (per 100 LA residents)					6.10	-3.12 to 15.32	5.05	-3.88 to 13.98	fixed-effect
LA – random intercept (sd)	29.25		24.50		20.56		20.58		random-effect

For peer review only - http://bmjopen.bmj.com/site/about/guidelines.xhtml

Time (financial year) – random slope (sd)	3.91		3.78		3.78		3.78		random-effect
Correlation between random intercept and random slope	0.14		-0.11		-0.45		-0.45		random-effect
Residual error	19.43		19.37		19.37		19.37		residual

ARA = adversity-related hospital admission; **LA** = local authority; **LSOA** = lower-layer super output area; **sd** = standard deviation.

For peer review only

For peer review only - http://bmjopen.bmj.com/site/about/guidelines.xhtml

Table A4 – Linear mixed-effect modelling results (Model 5)

Model parameter	Estimate	95% C.I.	Type of model parameter
Time (financial year) (where average maternal ARA = 0)	0.78	-0.54 to 2.10	fixed-effect
% of live births with maternal history of ARA (in 2006/07)	0.16	-1.93 to 2.25	fixed-effect
Effect of unit increase in time on average % of live births with maternal history of ARA (or vice versa)	0.44	0.15 to 0.72	fixed-effect
% of live births where mother < 20 years old	8.29	6.23 to 10.35	fixed-effect
% of live births where maternal postcode history within 10% most deprived LSOAs in England	0.06	-0.30 to 0.41	fixed-effect
% of live births where child has a complex chronic condition	14.19	5.37 to 23.01	fixed-effect
% of live births with low birth weight	0.60	-3.62 to 4.82	fixed-effect
LA population size (000s)	-0.08	-0.15 to -0.02	fixed-effect
% of dependent child households with lone parent	1.92	0.70 to 3.14	fixed-effect
Rate of violent crime (per 100 LA residents)	5.20	-3.86 to 14.25	fixed-effect
Local Authority – random intercept (sd)	18.55		random-effect
Residual error	21.35		residual

ARA = adversity-related hospital admission; **LA** = local authority; **LSOA** = lower-layer super output area; **sd =** standard deviation.

For peer review only - http://bmjopen.bmj.com/site/about/guidelines.xhtml

Table A5: Conditional coefficient estimates for percentage of live births with maternal history of adversity-related hospital admissions, by financial year (Model 5)

Measure	Financial year	Conditional coefficient estimate	95% C.I.
Effect of a 1% point increase in % of live births with maternal history of ARA in…	2006/07	0.13	-2.01 to 2.28
	2007/08	0.57	-1.32 to 2.48
	2008/09	1.01	-0.65 to 2.69
	2009/10	1.45	-0.03 to 2.93
	2010/11	1.89	0.59 to 3.21
	2011/12	2.33	1.13 to 3.53
	2012/13	2.77	1.61 to 3.93
	2013/14	3.21	2.03 to 4.39

ARA = adversity-related hospital admission.
Note: Created using R package 'interplot' https://cran.r-project.org/web/packages/interplot/index.html.

For peer review only - http://bmjopen.bmj.com/site/about/guidelines.xhtml

References

1. Bronfenbrenner U. The Ecology of Human Development. The Educational Forum. 1979.

2. Belsky J. Child maltreatment: An ecological integration. Am Psychol. 1980;

3. Sidebotham P. An ecological approach to child abuse: A creative use of scientific models in research and practice. Child Abus Rev. 2001;10(2):97–112.

4. Stith SM, Liu T, Davies LC, et al. Risk factors in child maltreatment: A meta-analytic review of the literature. Aggression and Violent Behavior. 2009.

5. Mulder TM, Kuiper KC, van der Put CE, Stams G-JJM, Assink M. Risk factors for child neglect: A meta-analytic review. Child Abuse Negl. 2018;77:198–210.

6. Sidebotham P, Heron J, Team AS. Child maltreatment in the “children of the nineties”: a cohort study of risk factors. Child Abuse Negl. 2006;

For peer review only

For peer review only - http://bmjopen.bmj.com/site/about/guidelines.xhtml

STROBE Statement—Checklist of items that should be included in reports of ***cohort studies***

	Item No	Recommendation	Reported on page
**Title and abstract**	1	(*a*) Indicate the study's design with a commonly used term in the title or the abstract	1
		(*b*) Provide in the abstract an informative and balanced summary of what was done and what was found	2
**Introduction**			
Background/rationale	2	Explain the scientific background and rationale for the investigation being reported	4
Objectives	3	State specific objectives, including any prespecified hypotheses	4
**Methods**			
Study design	4	Present key elements of study design early in the paper	4/5
Setting	5	Describe the setting, locations, and relevant dates, including periods of recruitment, exposure, follow-up, and data collection	5
Participants	6	(*a*) Give the eligibility criteria, and the sources and methods of selection of participants. Describe methods of follow-up	5-9
		(*b*) For matched studies, give matching criteria and number of exposed and unexposed	
Variables	7	Clearly define all outcomes, exposures, predictors, potential confounders, and effect modifiers. Give diagnostic criteria, if applicable	5-9
Data sources/ measurement	8*	For each variable of interest, give sources of data and details of methods of assessment (measurement). Describe comparability of assessment methods if there is more than one group	*5-9*
Bias	9	Describe any efforts to address potential sources of bias	5-9
Study size	10	Explain how the study size was arrived at	5-9
Quantitative variables	11	Explain how quantitative variables were handled in the analyses. If applicable, describe which groupings were chosen and why	5-10
Statistical methods	12	(*a*) Describe all statistical methods, including those used to control for confounding	10
		(*b*) Describe any methods used to examine subgroups and interactions	10
		(*c*) Explain how missing data were addressed	5-9
		(*d*) If applicable, explain how loss to follow-up was addressed	N/A
		(*e*) Describe any sensitivity analyses	N/A
**Results**			
Participants	13*	(a) Report numbers of individuals at each stage of study—eg numbers potentially eligible, examined for eligibility, confirmed eligible, included in the study, completing follow-up, and analysed	5
		(b) Give reasons for non-participation at each stage	N/A
		(c) Consider use of a flow diagram	appendix
Descriptive data	14*	(a) Give characteristics of study participants (eg demographic, clinical, social) and information on exposures and potential confounders	11-12
		(b) Indicate number of participants with missing data for each variable of interest	5-9

For peer review only - http://bmjopen.bmj.com/site/about/guidelines.xhtml

		(c) Summarise follow-up time (eg, average and total amount)	5
Outcome data	15*	Report numbers of outcome events or summary measures over time	11-12
Main results	16	(*a*) Give unadjusted estimates and, if applicable, confounder-adjusted estimates and their precision (eg, 95% confidence interval). Make clear which confounders were adjusted for and why they were included	12-13
		(*b*) Report category boundaries when continuous variables were categorized	N/A – no categorisation
		(*c*) If relevant, consider translating estimates of relative risk into absolute risk for a meaningful time period	N/A – AR reported
Other analyses	17	Report other analyses done—eg analyses of subgroups and interactions, and sensitivity analyses	13
**Discussion**			
Key results	18	Summarise key results with reference to study objectives	14
Limitations	19	Discuss limitations of the study, taking into account sources of potential bias or imprecision. Discuss both direction and magnitude of any potential bias	14
Interpretation	20	Give a cautious overall interpretation of results considering objectives, limitations, multiplicity of analyses, results from similar studies, and other relevant evidence	15
Generalisability	21	Discuss the generalisability (external validity) of the study results	14-15
**Other information**			
Funding	22	Give the source of funding and the role of the funders for the present study and, if applicable, for the original study on which the present article is based	19

*Give information separately for exposed and unexposed groups.

Note: An Explanation and Elaboration article discusses each checklist item and gives methodological background and published examples of transparent reporting. The STROBE checklist is best used in conjunction with this article (freely available on the Web sites of PLoS Medicine at http://www.plosmedicine.org/, Annals of Internal Medicine at http://www.annals.org/, and Epidemiology at http://www.epidem.com/). Information on the STROBE Initiative is available at http://www.strobe-statement.org.

For peer review only
